# Antibacterial Activity of GO-Based Composites Enhanced by Phosphonate-Functionalized Ionic Liquids and Silver

**DOI:** 10.3390/ma18081889

**Published:** 2025-04-21

**Authors:** Xinyu Liu, Xing Zhao, Hongda Qiu, Weida Liang, Linlin Liu, Yunyu Sun, Lingling Zhao, Xiao Wang, Hongze Liang

**Affiliations:** 1 Key Laboratory of Advanced Mass Spectrometry and Molecular Analysis of Zhejiang Province, School of Materials Science and Chemical Engineering, Ningbo University, Ningbo 315211, China; 15167214127@163.com (X.Z.); qiuhongda1998@163.com (H.Q.); liangweida94@gmail.com (W.L.); liulinlin1205@163.com (L.L.); zhaolingling@nbu.edu.cn (L.Z.); 2 Anhui Key Laboratory of Spin Electron and Nanomaterials of Anhui Higher Education Institutes, School of Chemistry and Chemical Engineering, Suzhou University, Suzhou 234000, China; sunyunyu2008@ahszu.edu.cn; 3 Health Science Center, Ningbo University, Ningbo 315211, China; wangxiao@nbu.edu.cn

**Keywords:** graphene oxide, phosphonate, functionalized ionic liquid, silver composite, antibacterial activity

## Abstract

The development of antibiotic-independent antimicrobial materials is critical for addressing bacterial resistance to conventional antibiotics. Currently, there is a lack of comprehensive understanding of ionic liquid-modified composites in antimicrobial applications. Here, we innovatively prepared GO-based composites modified with phosphonate ionic liquids via a series of surface functionalizations. The resulting antibacterial composites exhibit significant broad-spectrum activity against both Gram-negative and Gram-positive bacteria, including drug-resistant strains, with stronger efficacy against Gram-negative species. Additionally, the material features excellent long-term reusability and the ability to inhibit/destroy biofilms, which is vital for combating persistent infections. Mechanistic studies reveal its antibacterial effects through multiple pathways: disrupting bacterial membranes, inducing ROS, and inactivating intracellular substances—mechanisms less likely to promote resistance. Overall, these phosphonate ionic liquid-modified polycationic materials demonstrate substantial potential in treating bacterial infections, offering a promising strategy to tackle antibiotic resistance challenges.

## 1. Introduction

The problem of bacterial infections is a huge challenge for global public health [1]. Modern medicine greatly helps in dealing with infectious diseases and significantly increases healthy lifespan. However, the battle between mankind and bacteria has not yet ended. The efficacy of antibiotics in clinical applications has noticeably declined over time due to the prevalence of drug resistance (DR). Bacteria have developed over billions of years an efficient and adaptable defense system to protect themselves from any potential threats in the environment [2]. The misuse and abuse of antibiotics accelerate the development of drug-resistant strains, even leading to the emergence of multi-drug-resistant (MDR) strains [3]. The currently available antibiotics and their derivatives mainly rely on an established mechanism to exert antibacterial activity: disrupting cell wall synthesis, inhibiting protein synthesis, interfering with nucleic acid synthesis, disturbing metabolism, and damaging bacterial membranes. Due to the old mechanism, they are no longer as effective against DR bacterial strains as usual. Methicillin-resistant *Staphylococcus aureus* (MRSA) has emerged as the first “superbug”, causing bacteria to be highly insensitive to antibiotics, thereby leading to intractable infections [4]. The menace of Gram-negative bacterial strains to public health has been increasing due to DR [5]. Moreover, due to the presence of the outer membrane that serves as a barrier to drug permeation, the Gram-negative bacteria exhibit enhanced resistance to antibiotics compared to the Gram-positive bacteria, making these bacteria more difficult to kill [6]. To cope with these challenges, some tens of antibiotic candidates are investigated in clinical phases. However, most of them are derived from the old antibiotic scaffolds; thereby, limited opportunities can be expected to effectively overcome the problem of drug resistance [7]. Therefore, it is particularly important to explore new broad-spectrum and efficient therapeutic options that are different from traditional antibiotics.

In addition to striving to discover new antibiotics, non-antibiotic antimicrobial functional materials have received growing attention due to their minimal potential to cause drug resistance [8,9]. These materials mainly include natural antimicrobial materials (e.g., antimicrobial peptides (AMPs) [10], lysozyme [11], and chitosan (CS) [12]), and artificially synthesized antimicrobial materials (e.g., metal complexes [13] and metal-based nanoparticles [14], carbon-based materials [15], amine polymers [16], and ionic liquids (ILs) [17,18]). However, the above-reported materials have some drawbacks, such as low antimicrobial efficiency, non-durable antimicrobial effect, and low sustainability for continuous use. Therefore, composite materials that combine multiple antimicrobial components with multiple actions on bacteria have become a hot topic due to their broad-spectrum antibacterial activity [19], low risk of drug resistance [20], good biocompatibility [21], and long-lasting antimicrobial effects [22].

On the other hand, graphene oxide (GO) has been widely studied for its applications in materials science, energy storage and biomedicine due to its unique physicochemical properties. GO has a certain degree of antimicrobial activity, with mechanisms including mechanical wrapping, the destruction of bacterial cell membranes by its extremely sharp edges, and oxidative stress in bacteria [23]. However, the antimicrobial capacity of the pristine GO is limited, and further modification is required to enhance the antimicrobial properties. GO nanosheets, due to their high specific surface area and layered structure, as well as a large number of oxygen-containing functional groups, are very suitable as substrate materials for functional modification.

In this study, we report the design and synthesis of a novel graphene oxide-based composite, GO@PEI-PFIL-Ag^+^/Ag/AgBr, aiming to address the issue of stubborn bacterial infections through the diverse antibacterial mechanisms of its multiple components. This material integrates high-density cationic sites generated from the quaternization of polyethyleneimine by phosphonate esters, silver ions (Ag^+^), and silver/bromosilver (Ag/AgBr) nanoparticles, achieving a comprehensive effect of multiple actions, such as disrupting the bacterial cell membrane, inducing the generation of reactive oxygen species, and inhibiting intracellular metabolism. We hypothesized that this multi-component design would endow it with potent, rapid, and long-lasting antibacterial activity, especially against challenging pathogens like *P. aeruginosa* and MRSA. Experimental results confirm that the composite exhibits broad-spectrum antibacterial efficacy, the ability to inhibit and destroy biofilms, and maintains its performance over five reuse cycles, indicating its potential as a next-generation antibacterial agent that can address persistent and drug-resistant infections through rationally designed multi-mechanistic synergistic effects.

## 2. Materials and Methods

### 2.1. Materials and Reagents

All the reagents were from commercial sources and no further purification was required. Graphene was purchased from XFNANO, Inc. (Nanjing, China). Sulfuric acid (H_2_SO_4_), orthophosphoric (H_3_PO_4_) acid, and Triton X-100 were purchased from Shanghai Macklin Biochemical Technology Co., Ltd. (Shanghai, China). Potassium permanganate (KMnO_4_) was purchased from Wuxi Jiani Chemical Co., Ltd. (Wuxi, China). Sodium hydroxide (NaOH), hydrogen peroxide (H_2_O_2_, 30%), toluene, hydrobromic acid (HBr, 48 wt%), zinc sulfate heptahydrate (ZnSO_4_·7H_2_O), and silver nitrate (AgNO_3_) were purchased from Sinopharm Chemical Reagent Co., Ltd. (Shanghai, China). Hydrochloric acid (HCl) was purchased from Zhejiang Zhongxing Chemical Reagent Co., Ltd. (Jinhua, China). Polyethyleneimine (PEI, MW = 70 kDa, 50 wt% in H_2_O), glutaraldehyde (50 wt% in H_2_O), and crystal violet (98%) were purchased from Aladdin Chemical Reagent (Shanghai, China). Diethyl (3-bromopropyl) phosphonate was purchased from Meryer Chemical Technology Co., Ltd. (Shanghai, China). Anhydrous copper sulfate (CuSO_4_) was purchased from Shanghai Jinshan Tingxin Chemical Reagent Factory (Shanghai, China). Ferric sulfate (Fe_2_(SO_4_)_3_) was purchased from the Second Experimental Factory of Shanghai Shanhai Chemical and Industrial Group (Shanghai, China). Luria–Bertani (LB) and Mueller–Hinton (MH) media were purchased from Hangzhou Microbial Reagent Co., Ltd. (Hangzhou, China). Phosphate-buffered saline (PBS) was purchased from Sangon Biotech (Shanghai) Co., Ltd. (Shanghai, China). Deionized water was produced by a Milli-Q apparatus (Millipore, Bedford, MA, USA).

Gram-positive bacteria: reference *Staphylococcus aureus* (ATCC6538) and Methicillin-resistant *Staphylococcus aureus* (ATCC43300). Gram-negative bacteria: reference *Escherichia coli* (ATCC25922) and *Pseudomonas aeruginosa* (BNCC337940). All the aforementioned strains were preserved in our laboratory.

### 2.2. Characterization

Scanning electron microscopy (SEM) images and energy dispersive X-ray analysis (EDX) were recorded on a Nova NanoSEM 450 electron microscope (FEI, Hillsboro, OR, USA). Transmission electron microscopy (TEM) images were carried out on a JEM-1230 electron microscope (JEOL, Tokyo, Japan). Fourier-transform infrared spectroscopy (FT-IR) was taken on a NICOLET 6700 Fourier transform infrared spectrometer (Thermo Scientific, Waltham, MA, USA) using KBr pellets (Shimadzu, Kyoto, Japan). The X-ray photoelectron spectra (XPS) were performed by a Thermo Scientific K-Alpha spectrometer (Thermo Scientific, USA). The zeta potentials and nanoparticle sizes were measured on a Zetasizer Nano ZS90 analyzer (Malvern, Malvern, UK). Crystal information of samples was characterized by an D8 Advance X-ray diffractometer (Bruker, Ettlingen, Germany) with Cu Ka radiation. The Raman spectra were obtained using a DXR3 Raman Microscope instrument (Thermo Scientific, USA). Thermogravimetric analysis (TG) was obtained on the Q500 Thermogravimetric analyzer (TA, Westlake, OH, USA). Water contact angles were measured on a Dataphysics OCA25 (DataPhysics Instruments GmbH, Filderstadt, Germany) with a 3.0 μL water drop. UV–vis DRS spectra were recorded on a UV-3600i Plus (Shimadzu, Kyoto, Japan). Nucleic acid concentration was measured using the NanoDrop One Nucleic Acid Concentration Meter (Thermo Scientific, USA). Bacterial ROS levels were measured using an Infinite M200 Pro multifunctional full-wavelength enzyme labeler (Tecan, Mannedorf, Switzerland). Live/dead fluorescence staining and ROS images of bacteria were taken with an Olympus IX73 fluorescence microscope (Olympus, Tokyo, Japan).

### 2.3. Preparation of GO

The preparation was carried out by referring to Tour’s graphene oxide synthesis method [24]. Briefly, a mixture of concentrated H_2_SO_4_/H_3_PO_4_ (36:4 mL) with a volume ratio of 9:1 was slowly added to graphene (0.3 g, 1 wt% aliquot), and potassium permanganate (1.8 g) was carefully added in 6 equal parts to the mixture to produce a slight exothermic heat that should not exceed 35–40 °C, all of which were performed in an ice-water bath. The reaction was then heated to 50 °C and stirred for 12 h. The reaction was cooled to room temperature and slowly poured into a mixture containing 30% hydrogen peroxide (0.3 mL) on ice (40 mL). After centrifugation to remove the supernatant, the product was washed to neutrality with HCl and deionized water and dried overnight at room temperature under vacuum.

### 2.4. Preparation of GO@PEI

As mentioned earlier, GO@PEI was synthesized [25]. Briefly, 100 mg of the obtained graphene oxide was added to 80 mL of deionized water and ultrasonically dispersed, 5 g of PEI was dissolved in 100 mL of deionized water and ultrasonically dissolved, an aqueous GO dispersion was added to the PEI solution slowly drop by drop, and the reaction was carried out further for 12 h at 80 °C. The product (GO@PEI) was washed with deionized water and ethanol and freeze-dried under vacuum.

### 2.5. Preparation of GO@PEI-PFILOEt

The quaternization of GO@PEI is similar to that previously reported [26]. Briefly, GO@PEI (100 mg) and diethyl (3-bromopropyl) phosphonate (0.65 g) were added to anhydrous toluene (30 mL), dispersed by sonication for 30 min at room temperature before the reaction was refluxed at 85 °C for 16 h. After centrifugation, GO@PEI-PFILOEt was obtained and washed with ethyl acetate and dichloromethane, and dried in vacuum at 85 °C.

### 2.6. Preparation of GO@PEI-PFIL-Ag^+^/Ag/AgBr and GO@PEI-PFIL-M^n+^

A total of 100 mg of GO@PEI-PFILOEt was added to a hydrobromic acid solution (with a specific gravity of 24%), and the hydrolysis reaction was carried out at 115 °C for 2 h. Then, it was neutralized by the addition of sodium hydroxide solution (pH = 9). The obtained GO@PEI-PFIL-Na^+^ was washed several times with deionized water and ethanol, and dried under vacuum at 85 °C. A total of 25 mg of GO@PEI-PFIL-Na^+^ was, respectively, added to 8 mL of AgNO_3_, CuSO_4_, ZnSO_4_, and Fe_2_(SO_4_)_3_ solutions (0.1 mol/L), and incubated at room temperature for 2 h. Centrifugation was performed to obtain GO@PEI-PFIL-Ag^+^/Ag/AgBr, GO@PEI-PFIL-Cu^2+^, GO@PEI-PFIL-Zn^2+^, and GO@PEI-PFIL-Fe^3+^, which were then washed several times with deionized water and ethanol, and dried under vacuum conditions.

### 2.7. Minimum Inhibitory Concentration (MIC) Assay

The minimum inhibitory concentration of the composites was determined in 96-well plates using the micro broth dilution method [27]. Methicillin-resistant *Staphylococcus aureus* (ATCC43300) and *Staphylococcus aureus* (ATCC6538) were cultured using Mueller–Hinton (MH) medium, and *Escherichia coli* (ATCC25922) and *Pseudomonas aeruginosa* (BNCC337940) using Luria–Bertani (LB) medium. The strains were cultured at 37 °C until mid-logarithmic (OD600 = 0.5) and then diluted to a concentration of 1 × 10^5^ CFU/mL, and added to 96-well plates. The prepared samples were inoculated with a series of different concentrations of GO, GO@PEI, GO@PEI-PFILOEt, GO@PEI-PFIL-Cu^2+^, GO@PEI-PFIL-Zn^2+^, GO@PEI-PFIL-Fe^3+^, and GO@PEI-PFIL-Ag^+^/Ag/AgBr. The lowest concentration value that did not produce visible bacterial growth after incubation at 37 °C for 18–24 h was the MIC value. Each test was repeated three times.

### 2.8. Agar Diffusion Test

The antibacterial activity of different concentrations of GO@PEI-PFIL-Ag^+^/Ag/AgBr was tested by the agar diffusion method. The concentrations of *Escherichia coli*, *Pseudomonas aeruginosa*, *Staphylococcus aureus*, and Methicillin-resistant *Staphylococcus aureus* were diluted to 1 × 10^7^ CFU/mL. The bacterial suspension was spread evenly on Luria–Bertani (LB) agar plates using a sterile cotton swab. Sterile drug-sensitive tablets (6 mm in diameter) containing 20 μL of different concentrations of GO@PEI-PFIL-Ag^+^/Ag/AgBr and sterile PBS (control) were then carefully placed on the agar. Incubation was carried out overnight at 37 °C. The diameter of the zone of inhibition around the filter paper sheet was measured. Each experiment was repeated three times.

### 2.9. Short-Term Antibacterial Activity Test

Bacterial suspensions with a bacterial density of 1 × 10^7^ CFU/mL were inoculated with GO, GO@PEI, GO@PEI-PFILOEt, GO@PEI-PFIL-Cu^2+^, GO@PEI-PFIL-Zn^2+^, GO@PEI-PFIL-Fe^3+^, and GO@PEI-PFIL-Ag^+^/Ag/AgBr, respectively, and the PBS controls were prepared using the same method. The samples were incubated in a shaker at 37 °C for 2.5 h. Then, 100 μL dilutions of the samples at different multiplicities were evenly spread on LB plates and incubated at 37 °C for 24 h. Each set of experiments was repeated three times.

The colonies on the plates after incubation were counted, and the reduction rate of colonies was calculated as follows: (number of colonies in the control group − number of colonies in the medicated group)/number of colonies in the control group.

### 2.10. Long-Term Antibacterial Activity Test

GO@PEI-PFIL-Ag^+^/Ag/AgBr of 7.81 μg/mL and control samples were added to a bacterial suspension with a bacterial density of 1 × 10^7^ CFU/mL. Then, all the samples were allowed to stand at room temperature for one week, and afterwards, the samples were diluted in different multiples, and 100 μL of each sample was evenly spread on LB plates, and incubated at 37 °C for 24 h. The colonies on the plates were counted and the reduction rate of bacterial colonies in each group was recorded.

### 2.11. Time-Killing Curve Assay

The culture to mid-logarithmic stage was diluted to 1 × 10^7^ CFU/mL, followed by the addition of 1×, 2×, 4×, and 8 × MIC of GO@PEI-PFIL-Ag^+^/Ag/AgBr, with concentrations of 0.98, 1.95, 3.91, and 7.81 μg/mL in the *E. coli* group; and concentrations of 1.95, 3.91, 7.81, and 15.63 μg/mL in the *P. aeruginosa* group; concentrations in the *S. aureus* and MRSA groups were 7.81, 15.63, 31.25, and 62.5 μg/mL. The blank control group was treated with PBS. It was incubated at 37 °C, 250 rpm for 5 h, 100 μL of bacterial suspension was collected at half-hourly intervals, and the survival rate was calculated by using the plate colony counting method to determine the antimicrobial effect of GO@PEI-PFIL-Ag^+^/Ag/AgBr in different concentrations in treatment time. Each set of experiments was repeated three times.

### 2.12. Activity of GO@PEI-PFIL-Ag^+^/Ag/AgBr After Repeated Use

First, GO@PEI-PFIL-Ag^+^/Ag/AgBr at a concentration of 7.81 μg/mL was added to bacterial suspensions of *E. coli*, *P. aeruginosa*, *S. aureus*, and MRSA, each at a concentration of 1 × 10^7^ CFU/mL. The control group contained no GO@PEI-PFIL-Ag^+^/Ag/AgBr but had the same bacterial concentration. After 2.5 h of incubation, 100 μL of the mixture was diluted at different multiples, and bacterial reduction was calculated using the plate counting method. All the experiments were repeated three times. The solutions from both the experimental and control groups were then stored at room temperature for 24 h. For the second use, fresh bacterial suspensions were added to the previously used solutions of both groups [28], with no additional antibacterial material introduced. After another 2.5 h of treatment, 100 μL of the mixture was again diluted, plated, incubated, and analyzed for reduction rate. The third use was performed following the same procedure. In total, five replicate experiments were conducted to ensure reproducibility.

### 2.13. Hemolytic Test

Hemolysis of GO, GO@PEI, GO@PEI-PFILOEt, and GO@PEI-PFIL-Ag^+^/Ag/AgBr was assessed by measuring the amount of hemoglobin released from fresh human erythrocytes. Blood cells were washed 3 times with 10 mM PBS (pH = 7.4) and centrifuged 1500 rpm for 10 min at 25 °C. A total of 100 μL of erythrocytes (8%, *v*/*v*) and 100 μL of different concentrations of antimicrobial material were incubated for 1 h at 37 °C. After centrifugation at 1500 rpm for 5 min, the absorbance of the supernatant was measured at 540 nm. PBS and 0.1% Triton X-100 were used as negative and positive controls, respectively. Each experiment was repeated three times.

The hemolysis rate was calculated as follows: (OD value of treatment group − OD value of negative control group)/(OD value of positive control group − OD value of negative control group).

### 2.14. Cytotoxicity Assay

The effects of GO, GO@PEI, GO@PEI-PFILOEt, and GO@PEI-PFIL-Ag^+^/Ag/AgBr on the viability of mouse peritoneal RAW264.7 microphage cells were determined by using the Cell Counting Kit-8 (CCK-8) method [29]. Cells (2.5 × 10^4^ cells/wells) were added to 96-well plates and incubated in 5% (*v*/*v*) CO_2_/air environment at 37 °C overnight. The cell culture medium was discarded, and then culture with different concentrations of GO@PEI-PFIL-Ag^+^/Ag/AgBr (0.49, 0.98, 1.95, 3.91, 7.81, 15.63, 31.25, 62.5, and 125 μg/mL) and 7.81 μg/mL of GO, GO@PEI, and GO@PEI-PFILOEt was incubated for 24 h. Cells treated with an equal volume of PBS were used as a control group, and wells without added cells were used as a blank group. At the end of incubation, the supernatant was removed and washed with PBS three times, and then 100 μL of medium containing 10% WST-8 solution was added to each well. Incubation was performed at 37 °C under light protection for 0.5 h. The absorbance at 460 nm was measured. The experiment was repeated three times for each group.

The cell survival rate was calculated as follows: (OD value of treatment group − OD value of blank group)/(OD value of control group − OD value of blank group).

### 2.15. Inhibition and Destruction of Biofilm Assays

To evaluate the ability of these materials to inhibit the formation of bacterial biofilms, MRSA and *E. coli* were treated with various concentrations of GO@PEI-PFIL-Ag^+^/Ag/AgBr as well as the same concentrations of GO, GO@PEI, and GO@PEI-PFILOEt prior to biofilm formation.

Briefly, bacterial suspensions diluted with TSB medium (1 × 10^7^ CFU/mL) were added to a 96-well plate, followed by the addition of different concentrations of GO@PEI-PFIL-Ag^+^/Ag/AgBr and the same concentrations of GO, GO@PEI, and GO@PEI-PFILOEt to the bacterial suspension, and then cultured at 37 °C for 12 h to promote bacterial adhesion to the well walls. The group incubated with PBS was used as a control. After removing the medium, it was washed three times with PBS to wash away the planktonic bacteria. Biofilms were stained with 0.1% crystal violet solution (200 μL/wells) for 30 min. The excess staining was then washed off with PBS. Finally, the dye on the biofilm was dissolved in 95% ethanol (200 μL/wells), and the absorbance at 570 nm was measured with an enzyme marker. Each group was repeated three times.

The inhibition rate of biofilm was calculated as follows: (control group OD value − treatment group OD value)/control group OD value.

In order to evaluate the ability of materials to disrupt the formed biofilm, MRSA and *E. coli* (1 × 10^7^ CFU/mL) diluted in TSB medium were inoculated in a 96-well plate and cultured at 37 °C for 12 h to form biofilm. Unattached bacteria and medium were removed by rinsing with PBS, and the biofilm was treated with the different concentrations of GO@PEI-PFIL-Ag^+^/Ag/AgBr, GO, GO@PEI, and GO@PEI-PFILOEt, respectively. After incubating again for 12 h at 37 °C, the remaining biofilm in the wells washed with PBS, and then stained with 0.1% crystal violet solution (200 μL/well) for 30 min. The excess stain was washed away with PBS. Finally, the dye on the biofilm was dissolved in 95% ethanol (200 μL/wells), and the absorbance at 570 nm was measured with an enzyme marker. Each group was repeated three times.

The destruction rate of biofilm was calculated as follows: (control group OD value − treatment group OD value)/control group OD value.

### 2.16. SEM Characterization of Bacterial Cells

The bacteria *E. coli*, *P. aeruginosa*, and MRSA cultured to mid-logarithmic stage were incubated with 4 × MIC of GO@PEI-PFIL-Ag^+^/Ag/AgBr at 37 °C for 2.5 h and a control group was set up. Bacteria were collected by centrifugation and washed 3 times with 0.1 M PBS (pH = 7.2). The bacteria were then fixed with 2.5% glutaraldehyde at 4 °C for 15 min. After PBS washing twice, the bacteria were dehydrated with a gradient of ethanol (20–100%) for 5 min, and then air-dried overnight. Gold-palladium was sprayed on the surface of the samples, and the bacterial morphology was observed by a S4800 scanning electron microscope (Hitachi, Tokyo, Japan).

### 2.17. Live and Dead Bacteria Staining

Bacterial viability was detected using a commercial kit (SYTO9-PI Live and Dead Bacteria Stain Kit, Tjs. Bio, Thermo Fisher Scientific, Waltham, MA, USA). Bacteria were incubated with 4 × MIC of GO@PEI-PFIL-Ag^+^/Ag/AgBr for 2.5 h. Bacterial cells were collected by centrifugation, and stained with a mixture of SYTO 9 and propidium iodide (PI) at room temperature in the dark for 15 min. The fluorescence image was observed by using inverted fluorescence microscopy (Olympus, Tokyo, Japan).

### 2.18. Nucleotide Leakage Assay

Leakage of intracellular components of bacteria was assessed by detecting the concentration of nucleotides. The absorbance value at 260 nm (OD260) was measured to assess the concentration of nucleic acids [30]. Bacterial suspensions were treated with 1×, 2×, 4× and 8 × MIC of GO@PEI-PFIL-Ag^+^/Ag/AgBr and 7.81 μg/mL of GO, GO@PEI, GO@PEI-PFILOEt and GO@PEI-PFIL-Ag^+^/Ag/AgBr, and the control consisted of PBS-treated bacterial suspensions. After incubation at 37 °C for 2.5 h, the bacterial cell suspensions were filtered through a 0.22 μm sterile syringe filter membrane. Finally, the absorbance at 260 nm was measured.

### 2.19. Protein Leakage Assay

Leaked protein concentration in bacterial cells was determined using a commercial kit (Bradford Protein Concentration Assay Kit, Beyotime, Seoul, Republic of Korea). Bradford working solution was prepared according to the kit instructions. Bacterial cells were incubated overnight at 37 °C, then centrifuged (6000 rpm, 4 °C, 10 min) to collect the cells and washed 3 times with PBS. The bacterial suspension was treated with GO@PEI-PFIL-Ag^+^/Ag/AgBr at final concentrations of 1×, 2×, 4×, and 8 × MIC, as well as with GO, GO@PEI, and GO@PEI-PFILOEt at a concentration of 7.81 μg/mL. The control was PBS-treated bacterial suspension. After incubation at 37 °C for 2.5 h, the supernatant was collected by centrifugation (6000 rpm, 4 °C, 10 min), immediately freeze-dried under vacuum, incubated by adding Bradford’s working solution, and the absorbance at 562 nm was measured using an enzyme marker. The concentration of sample proteins was obtained from the plotted standard curve.

### 2.20. Determination of Cellular Total Reactive Oxygen Species (ROS)

The level of reactive oxygen species in bacterial cells was detected using a commercial kit (Reactive Oxygen Assay Kit, Beyotime). Briefly, bacteria with a final concentration of 10^8^ CFU/mL were collected by centrifugation, a dilution of 10 μM of DCFH-DA was added, and the cells were incubated for 20 min at 37 °C to load the probe. The excess unloaded DCFH-DA was then washed away, and the bacteria were stimulated by adding different concentrations of GO@PEI-PFIL-Ag^+^/Ag/AgBr for 1.5 h. The PBS control group was set up. The fluorescence intensity was detected using a multifunctional enzyme marker and photographed with an inverted fluorescence microscope with an excitation wavelength of 488 nm and emission wavelength of 525 nm.

### 2.21. Statistical Analysis

All data are presented as mean ± standard deviation (SD). A one-way analysis of variance (ANOVA) and post-hoc analysis (Tukey) were used for comparison between groups and multiple comparisons, respectively. Statistical significance was set at * *p* < 0.05, ** *p* < 0.01, and *** *p* < 0.001. All the experiments were repeated three times.

## 3. Results and Discussion

### 3.1. Synthesis of the GO@PEI-PFIL-Ag^+^/Ag/AgBr and GO@PEI-PFIL-M^n+^ Composites

The past decades have witnessed a growing interest in ILs, including the potential biomedical applications [18]. However, compared to the commonly available simple ILs, IL monomers and polymeric ILs, investigations on antibacterial activities and mechanisms of functionalized IL-based materials are relatively rare [17,18,31]. In recent years, we have reported our efforts on the applications of phosphonate (or phosphine oxide)-functionalized ionic liquids (PFILs) and the PFIL-based materials. These functional molecules and materials exhibit enhanced performances in the rare earth (RE) extraction [32], RE-based luminescence [33,34], lithium (ion) battery [35,36,37,38,39], and phosphopeptide enrichment [26,40,41]. On the other hand, the phosphate-modified biomolecules are well known and play important and indispensable roles in various biological processes in organisms [42]. These findings have greatly inspired studies on exploiting biomedical potential of organic phosphonates, structurally similar to phosphates, over the past decades [43]. For example, drugs of phosphonates have found successful applications in the treatment of various bone diseases [44]. Therefore, it could be expected that the enzymatically stable phosphonate-modified materials may possess intrinsic biocompatibility and show bioactivities by being involved in some biological events physically and chemically. GO nanosheets with a high specific surface area could be an ideal matrix to be functionalized to maximize the desired properties significantly in a cost-effective manner.

**Scheme 1:**
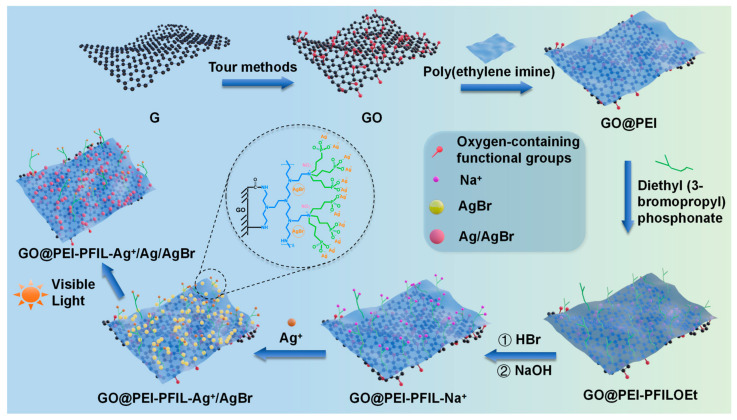
Schematic illustration of the synthetic procedure for preparation of GO@PEI-PFIL-Ag^+^/Ag/AgBr.

In this study, as a part of our work to explore drugs and materials with antibacterial activities [29,45,46], we report for the first time the preparation, antibacterial properties, and mechanism studies of the GO-based composites modified with phosphonate-functionalized ionic liquid (PFIL), which could be used to further chelate metal ions. The synthetic route of the GO@PEI-PFIL-Ag^+^/Ag/AgBr nanocomposite was illustrated in Scheme 1. First, the graphene sheets were oxidized to generate oxygen-containing functional groups on their surface and edges. Then, the amidation and addition of amino groups of PEI were carried out with respective hydroxyl and epoxy groups of GO covalently tethered PEI onto the surface of GO. The resulting GO@PEI was further reacted with excess diethyl (3-bromopropyl) phosphonate to quaternize N atoms of PEI, thereby conferring more positive charges on the PEI scaffold with concentrated phosphonate groups. The subsequent acid-catalyzed hydrolysis of phosphonate groups provided rich coordinating sites for metal ions. Finally, sodium salt of GO@PEI-PFIL-Na^+^ was reacted with silver nitrate to prepare GO@PEI-PFIL-Ag^+^/Ag/AgBr nanocomposite. Details of the chemical modifications involving amidation, quaternization, and hydrolysis are shown in Scheme S1. It should be noted that this resulting material contained three silver components: phosphonate-coordinated Ag (I) ions, deposited AgBr, and Ag nanocrystals resulting from the decomposition of AgBr exposed to sunlight. For comparison, GO@PEI-PFIL-Cu^2+^, GO@PEI-PFIL-Zn^2+^, and GO@PEI-PFIL-Fe^3+^ nanocomposites were prepared similarly by using corresponding metal salts in the metal immobilization step. As a result, such composites are highlighted by the unique combination of bactericidal components: (1) high surface area GO matrix available for modification, (2) high density of positive charges by quaternization of PEI, and (3) high density of phosphonate groups available for metal binding. The unique feature of these composites might harbor enhanced antimicrobial potency and less potential to develop drug resistance by multiple models of actions (such as ROS induction, membrane damage, etc.) [47,48,49,50,51,52] on bacteria.

### 3.2. Characterization of the GO@PEI-PFIL-Ag^+^/Ag/AgBr Nanocomposite

**Figure 1:**
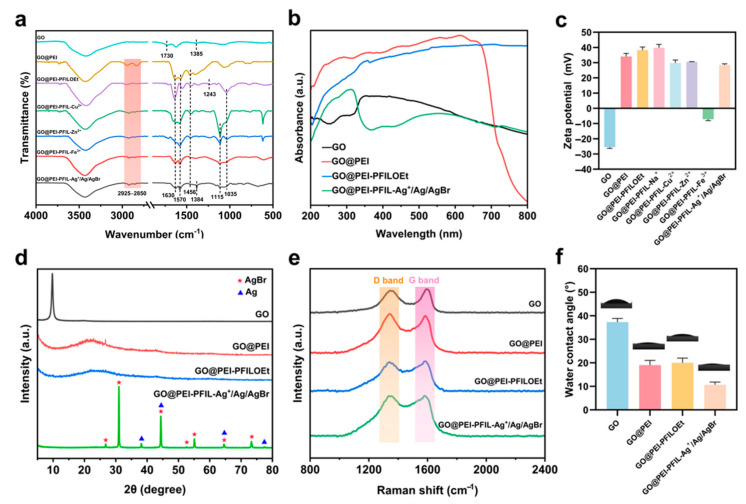
(**a**) FTIR spectra of GO, GO@PEI, GO@PEI-PFILOEt, GO@PEI-PFIL-Cu^2+^, GO@PEI-PFIL-Zn^2+^, GO@PEI-PFIL-Fe^3+^, and GO@PEI-PFIL-Ag^+^/Ag/AgBr. (**b**) UV–vis DRS spectra of GO, GO@PEI, GO@PEI-PFILOEt, and GO@PEI-PFIL-Ag^+^/Ag/AgBr. (**c**) Bar graph of zeta potentials of GO, GO@PEI, GO@PEI-PFILOEt, GO@PEI-PFIL-Na^+^, GO@PEI-PFIL-Cu^2+^, GO@PEI-PFIL-Zn^2+^, GO@PEI-PFIL-Fe^3+^, and GO@PEI-PFIL-Ag^+^/Ag/AgBr. (**d**) XRD patterns of GO, GO@PEI, GO@PEI-PFILOEt, GO@PEI-PFIL-Ag^+^/Ag/AgBr, and AgBr. (**e**) Raman spectroscopy of GO, GO@PEI, GO@PEI-PFILOEt, and GO@PEI-PFIL-Ag^+^/Ag/AgBr. (**f**) Water contact angle of GO, GO@PEI, GO@PEI-PFILOEt, and GO@PEI-PFIL-Ag^+^/Ag/AgBr.

The FTIR spectra of GO, GO@PEI, GO@PEI-PFILOEt, GO@PEI-PFIL-Cu^2+^, GO@PEI-PFIL-Zn^2+^, GO@PEI-PFIL-Fe^3+^, and GO@PEI-PFIL-Ag^+^/Ag/AgBr are illustrated in Figure 1a. The FTIR results indicate that after the graft of PEI, two new absorption peaks are observed at ~2850 and 2925 cm^−1^ in the FTIR spectra of GO@PEI, GO@PEI-PFILOEt, GO@PEI-PFIL-M^n+^, and GO@PEI-PFIL-Ag^+^/Ag/AgBr, which are assignable to the symmetric and asymmetric stretching modes of methylene (-CH_2_-) groups in PEI [53]. Additionally, the C=O (1730 cm^−1^) vibration of the carboxylic group observed in GO, shifted to 1630 cm^−1^ in GO@PEI, GO@PEI-PFILOEt, GO@PEI-PFIL-M^n+^, and GO@PEI-PFIL-Ag^+^/Ag/AgBr, indicating the success of amidation between the amino groups of PEI and the carboxylic groups of GO [53]. Adsorptions at 1456 cm^−1^ (stretching vibration of the C-N bond) and 1570 cm^−1^ (deformation vibration of N-H bond) can be observed, which give an additional support to the amide formation. The characteristic absorption band (1243 cm^−1^) of the P=O group can be found in GO@PEI-PFILOEt. After the hydrolysis of GO@PEI-PFILOEt and formation of ionic bonds with different metal ions, the absorption peak of the P=O group can be observed at 1115 cm^−1^, which also proves its successful hydrolysis and binding with different metal ions. The peak at 1035 cm^−1^, attributable to the typical vibration of P-O stretching, is observed in GO@PEI-PFILOEt, GO@PEI-PFIL-M^n+^, and GO@PEI-PFIL-Ag^+^/Ag/AgBr. An additional absorption at 1384 cm^−1^ corresponding to nitrate anion in GO@PEI-PFIL-Ag^+^/Ag/AgBr is observed, indicating that nitrate serves as the counterbalancing anion after immobilization with AgNO_3_ and the deposition of AgBr.

As shown in Figure 1b, the optical absorption properties of GO, GO@PEI, GO@PEI-PFILOEt, and GO@PEI-PFIL-Ag^+^/Ag/AgBr were detected using UV–visible diffuse reflectance spectroscopy. GO exhibits absorption in the visible light region. After being modified to GO@PEI, the composite material extends its absorption capability to the ultraviolet region. With further functionalization, the absorption range of GO@PEI-PFILOEt also spans across the whole UV–vis region. As for GO@PEI-PFIL-Ag^+^/Ag/AgBr, besides the significant absorption of silver bromide in the ultraviolet region, a dune-shaped absorption was observed in the visible region of 370–800 nm. This proves the presence of metallic silver particles in the composite material, which can generate surface plasmon resonance (SPR) absorption in the visible light region [54,55]. Under sunlight, AgBr forms Ag/AgBr heterojunctions, which under photocatalytic action, generate electron–hole pairs. The photoelectrons generated in the conduction band (CB) of AgBr promote the production of reactive oxygen species (ROS), which greatly enhance the antibacterial activity of the composite [56].

To further verify the successful fabrication of GO@PEI-PFIL-Ag^+^/Ag/AgBr, all intermediates in ethanol were subjected to the zeta potential test (Figure 1c). The first observable change in zeta potential from −25.57 mV to +34.07 mV was noticed upon modification with PEI, verifying the presence of the vast number of positively charged nitrogen-containing groups. After quaternization, the zeta potential of GO@PEI-PFILOEt increased to +38.27 mV, reflecting the net result of the cationic nature of ionic liquid and phosphonate groups. A further increase in the zeta potential was observed after the hydrolyzation and neutralization of phosphonate with NaOH (+39.73 mV for GO@PEI-PFIL-Na^+^). After immobilization with Cu^2+^, Zn^2+^, and Fe^3+^, the final potential ended with +29.80 mV, +30.53 mV, and −7.21 mV, respectively. The zeta potential of GO@PEI-PFIL-Ag+/Ag/AgBr is +28.47 mV, revealing the difference in the interaction between negatively charged phosphonates and respective cationic Ag^+^, Cu^2+^, Zn^2+^, and Fe^3+^ ions. Additionally, the increased electropositivity of the material surface enhances the electrostatic interaction with negatively charged bacterial cell wall and membranes.

The XRD spectra of GO, GO@PEI, GO@PEI-PFILOEt, and GO@PEI-PFIL-Ag^+^/Ag/AgBr are shown in Figure 1d. For GO, a new peak centered at 2θ = 9.7° is observed, which corresponds to an interlayer spacing of (002) of about 0.907 nm according to Bragg’s equation, and may be due to the high degree of exfoliation and structural disorder in GO. The interlayer spacing of GO depends on the number of oxygen-containing groups on the surface of the material, as well as the number of water molecules absorbed with neighboring GO flakes. After modification with PEI and diethyl phosphonate, broad peaks were observed in the GO@PEI and GO@PEI-PFILOEt composites, which could be related to the amorphous diffraction interfered by PEI and diethyl phosphonate. GO@PEI exhibits a very weak broad peak with 2θ appeared at approximately 21.9°, which shifts to ≈21.5° in GO@PEI-PFILOEt, indicating a slight increase in the interlayer spacing of the composite material. The XRD results of GO@PEI-PFIL-Ag^+^/Ag/AgBr show intensive and distinct diffraction peaks. The diffraction peaks marked with pentagrams at approximately 2θ = 26.7° (111), 30.9° (200), 44.3° (220), 52.5° (311), 55.0° (222), 64.5° (400), and 73.2° (420) are attributed to the typical characteristic peaks of silver bromide (JCPDS no. 06-0438), while the peaks marked with triangles at approximately 2θ = 38.2° (111), 44.3° (200), 64.5° (220), and 77.9° (311) exhibit the typical diffraction peaks of metallic silver (JCPDS no. 04-0783). All the above observations confirm the presence of concentrated silver and silver bromide nanoparticles, which dramatically suppress the detection of the sharp peak (9.7°) observed in GO and broad peaks in GO@PEI and GO@PEI-PFILOEt composites.

The Raman spectra are shown in Figure 1e, where GO, GO@PEI, GO@PEI-PFILOEt, and GO@PEI-PFIL-Ag^+^/Ag/AgBr present two characteristic bands, D and G. The emergence of the D band is due to defects and disorder in the sp^2^ hybridized structure of carbon atoms, while the G band is associated with in-plane vibrations of sp^2^ carbon atoms [57]. The values of D and G bands as well as the I_D_/I_G_ ratios for all intermediates and the target nanocomposite were listed in Table S1. The D and G bands are centered at 1348 and 1596 cm^−1^ for GO, 1343 and 1585 cm^−1^ for GO@PEI, and 1340 and 1581 cm^−1^ for GO@PEI-PFILOEt. The characteristic bands for GO@PEI-PFIL-Ag^+^/Ag/AgBr are located at 1348 and 1578 cm^−1^. The increased I_D_/I_G_ ratio from 0.94 (GO) to 1.05 (GO@PEI) indicates the reaction of active oxygen-containing functional groups of GO, due to the chemical bond formation between oxygen-containing groups of GO and amino groups of PEI. The I_D_/I_G_ ratio of GO@PEI-PFILOEt is 0.97, indicating that the introduction of the phosphonate ester group reduced the defects in the material. The I_D_/I_G_ ratio of GO@PEI-PFIL-Ag^+^/Ag/AgBr is 0.98, which is almost consistent with that of GO@PEI- PFILOEt, indicating that the introduction of silver ions and Ag/AgBr nanoparticles did not significantly affect the material’s structure.

The contact angles of GO, GO@PEI, GO@PEI-PFILOEt, and GO@PEI-PFIL-Ag^+^/Ag/AgBr were measured as shown in Figure 1f. Modification with PEI made the contact angle change from 37° (pristine GO) to 19° due to the instruction of more hydrophilic amino groups. Further quaternization with bromoalkylphosphonate slightly increased the contact angle of GO@PEI-PFILOEt to 20°. It is reasonable that the formation of numerous hydratable ammonium salts offsets the influence of the hydrophobic diethyl phosphonates. After the hydrolysis of phosphonates and metal immobilization, the resulting GO@PEI-PFIL-Ag^+^/Ag/AgBr nanoparticle is more hydrophilic with a contact angle of 10.67°. These changes indicate that the prepared GO@PEI-PFIL-Ag^+^/Ag/AgBr has excellent hydrophilicity. Bacteria generally prefer to adhere to hydrophobic surfaces rather than hydrophilic ones, as hydrophobic surfaces can provide a higher adhesive force for bacteria [58]. Therefore, modifying the hydrophilicity of material surfaces can reduce the adsorption of bacteria and extracellular polymeric substances (EPS), delay the formation of biofilms, and further kill bacteria in combination with the antimicrobial components in the material.

**Figure 2:**
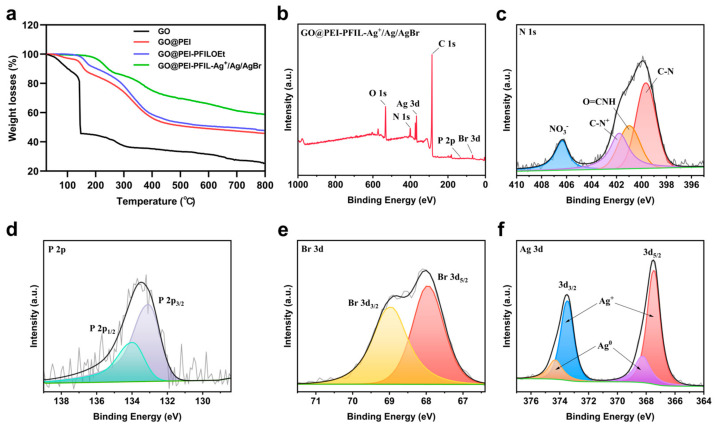
(**a**) Thermalgravimetric curves of GO, GO@PEI, GO@PEI-PFILOEt, and GO@PEI-PFIL-Ag^+^/Ag/AgBr. (**b**) XPS survey spectra of GO@PEI-PFIL-Ag^+^/Ag/AgBr. XPS spectra of N 1s (**c**), P 2p (**d**), Br 3d (**e**), and Ag 3d (**f**) of GO@PEI-PFIL-Ag^+^/Ag/AgBr.

The thermogravimetric behavior of GO, shown in Figure 2a, was consistent with the reported [59]. The mass loss at lower than 150 °C could be attributed to the release of the adsorbed water. The subsequent sharp loss was related to the pyrolysis of the most unstable oxygen-containing functional groups of GO, whereas the mass loss of more stable oxygen-containing functional groups slowed down in the interval from 300–800 °C. Compared with GO, the reduction by 35% for GO@PEI from 170 °C to 410 °C was ascribed to the decomposition of PEI [60], indicating that there is a large amount of PEI anchored on the surface of GO. The decomposition of GO@PEI-PFILOEt is roughly similar to that of GO@PEI, with a slight reduction in the weight loss. The decomposed curve of GO@PEI-PFIL-Ag^+^/Ag/AgBr shows a certain change with less mass loss due to the uptake of silver species.

The chemical composition of GO@PEI-PFIL-Ag^+^/Ag/AgBr was investigated using x-ray photoelectron spectroscopy (XPS). As shown in Figure 2b, the presence of the elements carbon, oxygen, nitrogen, phosphorus, bromine, and silver in GO@PEI-PFIL-Ag^+^/Ag/AgBr was confirmed, and the respective atomic contents of each were 79.36%, 11.79%, 6.23%, 0.45%, 0.92%, and 1.25% (Table S2). In addition, the peaks at 399.6, 400.9, 401.7, and 406.3 in the N 1s spectrum in Figure 2c correspond to the nitrogen atoms in the PEI, O=C-NH, C-N^+^, and NO_3_^–^, respectively [61,62], verifying that PEI was covalently grafted onto the GO by amide bonds, and was successfully quaternized by diethyl(3-bromopropyl)phosphonate. Meanwhile, bromides were replaced by nitrates as counter anions in the silver immobilization process with silver nitrate, thus leading to the generation of AgBr and Ag nanoparticles. The successful modification was further demonstrated by the peaks at 284.1 (C-P), 285.4 (C-N), and 288.3 (O=CN) of the C 1s spectrum in Figure S1 [63,64]. In Figure 2d, the P 2p_3/2_ and P 2p_1/2_ peaks at 133.1 and 134.0 demonstrate the presence of phosphorus in the form of phosphonate. The presence of silver/silver bromide is identified in Figure 2d,e, where the Br 3d spectrum clearly shows a peak with a composition of two overlapping spin-orbital components (∆ ≈ 1 eV) of 67.9 (Br 3d_5/2_) and 68.9 (Br 3d_3/2_). In the Ag 3d region, there are two peaks due to the presence of Ag 3d_5/2_ and Ag 3d_3/2_, which can be further divided into sub-peaks at 367.5, 368.3, 373.5, and 374.3 eV, respectively. The sub-peaks at 367.5 and 373.5 eV are attributed to Ag^+^, while the sub-peaks at 368.3 and 373.4 eV belong to Ag^0^, indicating that the silver in GO@PEI-PFIL-Ag^+^/Ag/AgBr exists in two oxidation states, which confirms the successful formation of Ag/AgBr nano-mixed structures in the composite material.

**Figure 3:**
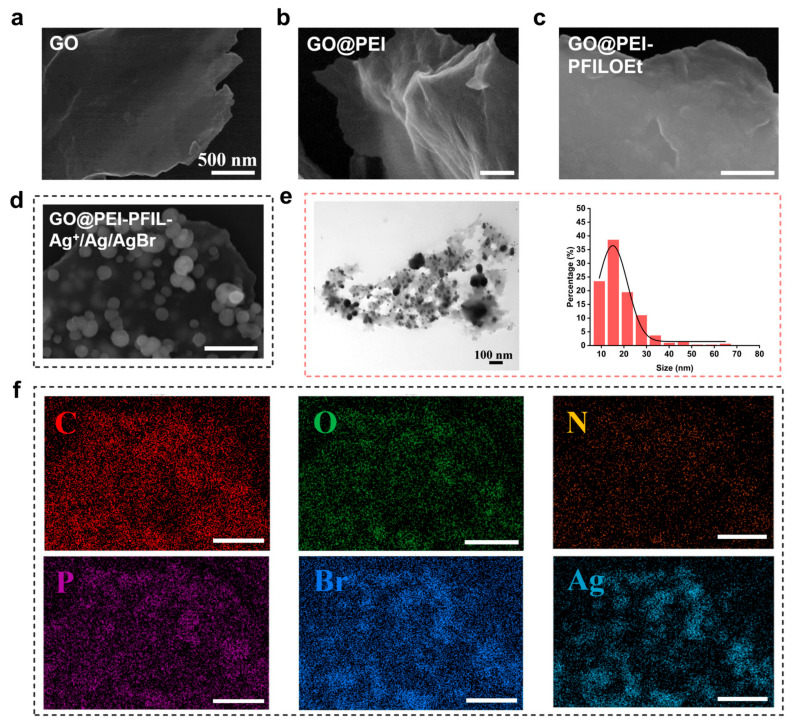
SEM analysis of GO (**a**), GO@PEI (**b**), GO@PEI-PFILOEt (**c**), and GO@PEI-PFIL-Ag^+^/Ag/AgBr (**d**). (**e**) TEM image of GO@PEI-PFIL-Ag^+^/Ag/AgBr, and size distribution of silver ions and silver bromide nanoparticles. (**f**) Elemental mapping of GO@PEI-PFIL-Ag^+^/Ag/AgBr, including C, O, N, P, Br, and Ag elements. Except for Figure 3e, the scale of all other figures is 500 nm.

The morphological studies on stepwise surface modification can provide more support for the success of material functionalization. Upon the grafting of PEI, the profile of the flake-like structure of GO looked like a steep mountain covered with a layer of snow, as illustrated in Figure 3b, and the subsequent quaternization with diethyl (3-bromopropyl) phosphonate reshaped it into a sandy landscape (Figure 3c). The elemental mappings and energy dispersive X-ray spectroscopy (EDS) results of GO, GO@PEI, and GO@PEI-PFILOEt are shown in Figure S2 and Table S3, indicating that surface modifications took place evenly. Figure 3d of GO@PEI-PFIL-Ag^+^/Ag/AgBr presents a strikingly different appearance with scattered Ag/AgBr nanoparticles, clearly indicating the achievement of silver immobilization and deposition. For comparison, the analysis of SEM and EDS of GO@PEI-PFIL-Cu^2+^ is presented in Figure S3. The immobilized Cu could be found in 2.60 atom%, and no nanoparticles were observed. These results, as well as the aforementioned XRD of GO@PEI-PFIL-Ag^+^/Ag/AgBr (Figure 1d), proved that the observed nanoparticles (Figure 3d) could be assigned to the metallic silver and AgBr. Further detection of C, O, N, P, Br, and Ag elemental contents and compositions in GO@PEI-PFIL-Ag^+^/Ag/AgBr by elemental mappings (Figure 3f) and EDS (Figure S4) demonstrated the successful functionalization modification of the composites. The stacked laminar structure of GO@PEI-PFIL-Ag^+^/Ag/AgBr was also exhibited by TEM (Figure 3e), and the size distribution of nanoparticles scattered on the surface was measured to be in the approximate range of 10–20 nm by counting.

### 3.3. Antimicrobial Activity of Nanocomposites

#### 3.3.1. Antimicrobial Evaluation by MIC and Inhibition Zone Assays

The antimicrobial activities were assayed first by using the micro broth dilution method. Minimum inhibitory concentration (MIC) is generally recognized as one of the most basic laboratory measurements for evaluating the antimicrobial efficacy of an antimicrobial agent, with lower MIC values indicating better action against bacteria. The MIC values of GO, GO@PEI, GO@PEI-PFILOEt, GO@PEI-PFIL-Cu^2+^, GO@PEI-PFIL-Zn^2+^, GO@PEI-PFIL-Fe^3+^, and GO@PEI-PFIL-Ag^+^/Ag/AgBr against Gram-negative bacteria (*E. coli*, *P. aeruginosa*) and Gram-positive bacteria (MRSA, *S. aureus*) are shown in Table 1. The naked GO, decorated GO@PEI, and GO@PEI-PFILOEt did not show detectable activity at concentrations lower than 250 μg/mL. It has been reported that graphene oxide shows antimicrobial effects at higher concentrations, and the activity is dependent on surface morphology, size, concentration, and contact time [65]. The higher MIC values (>250 μg/mL) of GO@PEI and GO@PEI-PFILOEt by the broth dilution method might be due to the aggregation of the composites during the experimental process, resulting in insufficient contact with the bacterial cells, and did not fully reflect their antimicrobial potentials.

**Figure 4:**
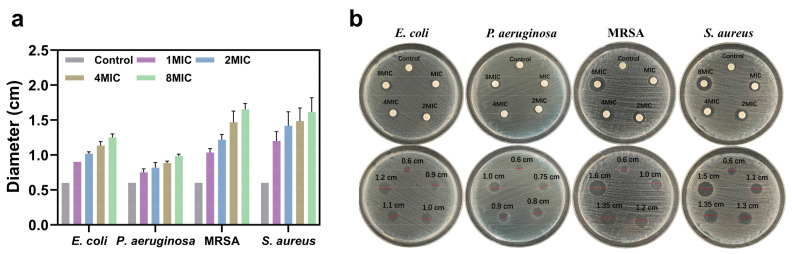
(**a**) Inhibitory zone diameter of GO@PEI-PFIL-Ag^+^/Ag/AgBr for *E. coli*, *P. aeruginosa*, MRSA, and *S. aureus*. (**b**) Digital images showing the inhibition halos caused by GO@PEI-PFIL-Ag^+^/Ag/AgBr against *E. coli*, MRSA, *P. aeruginosa*, and *S. aureus*.

After the immobilization of metal ions, GO@PEI-PFIL-Cu^2+^, GO@PEI-PFIL-Zn^2+^ and GO@PEI-PFIL-Fe^3+^ showed increased activities against some tested bacterial strains, indicating a beneficial effect of the introduction of metal ions. Of the seven materials experimented on, GO@PEI-PFIL-Ag^+^/Ag/AgBr composite exhibited the strongest potency against all the tested strains. The MIC values against *E. coli*, *P. aeruginosa*, MRSA, and *S. aureus* were ≤0.98 μg/mL, ≤1.95 μg/mL, ≤7.81 μg/mL, and ≤7.81 μg/mL, respectively, under experimental conditions. The antimicrobial efficacy of GO@PEI-PFIL-Ag^+^/Ag/AgBr composite is superior or comparable to other reported GO-based and silver-based antibacterial materials [56,66,67,68,69,70,71,72,73]. The antibacterial properties of several antibacterial materials based on graphene oxide or silver were compared, as shown in Table S4. The best performance of GO@PEI-PFIL-Ag^+^/Ag/AgBr is likely due to the combination of the following antibacterial factors. Firstly, it was reported that biocompatible PEI showed some antibacterial activity due to strong electrostatic interactions between amino groups and bacterial cell membranes, leading to bacterial death [74]. Secondly, PEI-based ionic liquid generated by the quaternization of PEI with bromoalkylphosphonate offered a high density of cations, which can interact with the surface of bacterial cells, causing irreversible damage to the bacterial cell membrane [75,76]. Thirdly, highly concentrated silver species were realized by phosphonate coordination, AgBr deposition, and in situ generated metallic Ag by the photo-induced decomposition of AgBr. Metal ions are widely recognized for their potent, broad-spectrum antimicrobial properties and their reduced propensity to induce microbial resistance. Among these ions, silver ions stand out for their superior antimicrobial efficacy. Silver ions indirectly produce reactive oxygen groups (ROS) [77], causing bacteria to lose a range of normal functions, and can also disrupt cell membrane integrity [78]. Silver/silver halide (Ag/AgX, where X = Cl, Br, I) nanoparticles, due to their ability to effectively generate reactive oxygen species (ROS) under visible light irradiation, have demonstrated excellent photocatalytic disinfection capabilities against pathogens under light conditions [79]. In addition, it was also evident by agar diffusion experiments (Figure 4a,b) that the diameter of the inhibition zone becomes longer as the concentration of the material increases, which indicates that the antimicrobial ability of GO@PEI-PFIL-Ag^+^/Ag/AgBr has a concentration-dependent effect. The higher the concentration, the stronger the antimicrobial activity of the material.

It has been found that the coordination chemistry of the ligands exhibits a profound impact on the antibacterial activity of Ag(I) complexes [80]. According to the hard–soft-acid-base (HSAB) theory, the weak bonding of Ag-O (carboxylate) would release Ag(I) ions more easily than the strong bonding of Ag-P and Ag-S, and thus facilitate the ligand exchange with O-, N-, and S-donors of biological ligands (DNA, proteins, enzymes, and membranes) [81], leading to the death of bacteria. In our case, phosphonate-coordinated Ag(I) ions would dissociate more easily than those coordinated by carboxylates. Therefore, GO@PEI-PFIL-Ag^+^/Ag/AgBr exhibits better and faster antibacterial effectiveness. In addition, the phosphonate groups in GO@PEI-PFIL-Ag^+^/Ag/AgBr may help transport the coordinated Ag(I) ions into Gram-positive bacterial cells via sequential ligand exchange with wall teichoic acids (WLAs) and lipoteichoic acids (LTAs) [82,83], due to the comparable metal coordination ability between phosphate and phosphonate groups. It could be noted that GO@PEI-PFIL-Ag^+^/Ag/AgBr is more potent against Gram-negative bacteria than Gram-positive bacteria (Table 1). This difference in antibacterial activities between Gram-negative and Gram-positive bacteria may originate from the difference in cell envelopes. The thinner peptidoglycan layer in the cell wall of the Gram-negative bacteria could not efficiently retard penetration of metal ions [84].

#### 3.3.2. Short-Term and Long-Term Antimicrobial Activity

**Figure 5:**
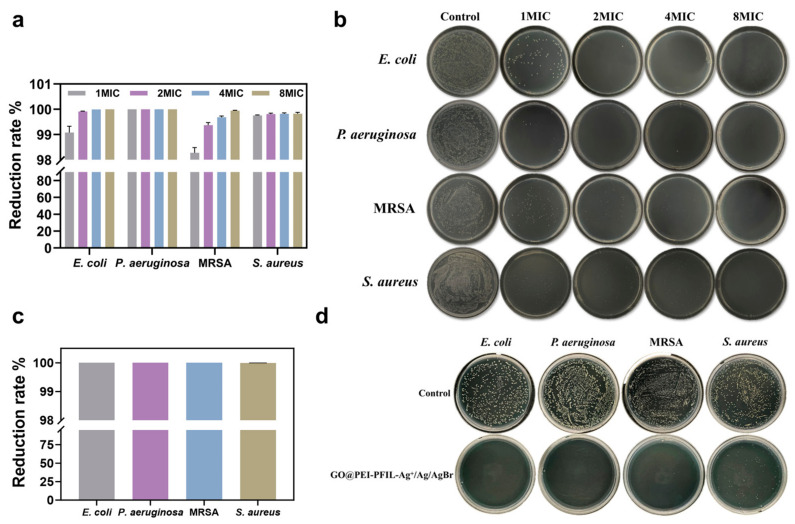
(**a**) Reduction percentage of bacteria treated with GO@PEI-PFIL-Ag^+^/Ag/AgBr for 2.5 h. (**b**) Bacterial colonies on LB plates of *E. coli*, MRSA, *P. aeruginosa*, and *S. aureus* treated with GO@PEI-PFIL-Ag^+^/Ag/AgBr for 2.5 h. (**c**) Reduction percentage of bacteria treated with GO@PEI-PFIL-Ag^+^/Ag/AgBr for one week. (**d**) Digital images of bacterial colonies on LB plates after *E. coli*, *P. aeruginosa*, MRSA, and *S. aureus* treated with GO@PEI-PFIL-Ag^+^/Ag/AgBr for one week.

We determined the short-term antimicrobial activity in order to further evaluate the antimicrobial ability of the nanocomposites. The CFU counting method was used to evaluate the antimicrobial activity of GO@PEI-PFIL-Ag^+^/Ag/AgBr on *E. coli*, *P. aeruginosa*, MRSA, and *S. aureus* strains for short-term antimicrobial properties. The results of the experiments were expressed using the reduction rate by calculating the relative values of bacterial colony counts after treatment with different concentrations of GO@PEI-PFIL-Ag^+^/Ag/AgBr to those of the control PBS treatment. The experiments were performed at a shaking speed of 250 rpm, allowing the material to be well dispersible to keep good contact with bacteria. As shown in Figure 5a, GO@PEI-PFIL-Ag^+^/Ag/AgBr exhibited a strong potency against all the tested bacterial strains within 2.5 h. Even at a concentration of 1 × MIC, the reduction rates were up to 99.1% (*E. coli*), 100% (*P. aeruginosa*), 98.3% (MRSA), and 99.8% (*S. aureus*), respectively. It was revealed that GO@PEI-PFIL-Ag^+^/Ag/AgBr could inhibit the growth of Gram-negative strains much faster and more efficiently than Gram-positive strains. The difference in efficiency can be visualized by the images of plates exhibited in Figure 5b. In order to distinguish the impact of each structural modification on the antimicrobial activities against the four tested strains, we conducted more experiments at a concentration of 2 × MIC (based on GO@PEI-PFIL-Ag^+^/Ag/AgBr). As shown in Figure S5, in comparison with the parental GO, polymeric ionic liquid GO@PEI-PFILOEt, generated by quaternization on PEI, exhibited a significant reduction rate of bacterial growth of *S. aureus* (95.1%), drug-resistant MRSA (97.1%), and intractable *P. aeruginosa* (86.7%). These promising results definitely verified that the introduction of phosphonate-modified ionic liquid could dramatically enhance the antibacterial activity. IL-based materials recently have attracted increasing attention due to their antimicrobial activities. Although long alkyl chains can increase antibacterial potency, the risk of cytotoxicity is also increased [17]. Our study demonstrates that the introduction of a phosphonate-functionalized ionic component can endow materials with excellent antibacterial potency without causing cytotoxicity by the usage of long lipophilic chains. These metal-free IL-based materials can exert their antibacterial activity without the problem of metal-induced toxicity. Metals in these composites play varied roles. Ag-containing composite always showed the best activity against all the strains, while others behaved quite differently, depending on the bacterial strains examined. For example, compared to GO@PEI-PFILOEt, GO@PEI-PFIL-Cu^2+^, GO@PEI-PFIL-Zn^2+^, and GO@PEI-PFIL-Fe^3+^ exhibited decreased potency against *P. aeruginosa* and *S. aureus*, while they showed some increased or equal activities against *E. coli* and MRSA.

The ability to inhibit bacterial growth over a long period of time is one of the most important properties for antimicrobial materials. As shown in Figure 5c,d, GO@PEI-PFIL-Ag^+^/Ag/AgBr effectively kills the bacteria and sustains the inhibition for at least one week, even in nutrient-rich environments, demonstrating a stable and powerful bacterial inhibition effectiveness. The survival rate of all four bacteria after treatment with the same concentration of GO@PEI-PFIL-Ag^+^/Ag/AgBr was less than 0.01%. Comparing the short-term antimicrobial effects, GO@PEI-PFIL-Ag^+^/Ag/AgBr showed little or no reduction in the antimicrobial effects against the four bacteria, maintaining its excellent antimicrobial properties.

#### 3.3.3. Bactericidal Kinetics and Reusability of GO@PEI-PFIL-Ag^+^/Ag/AgBr

**Figure 6:**
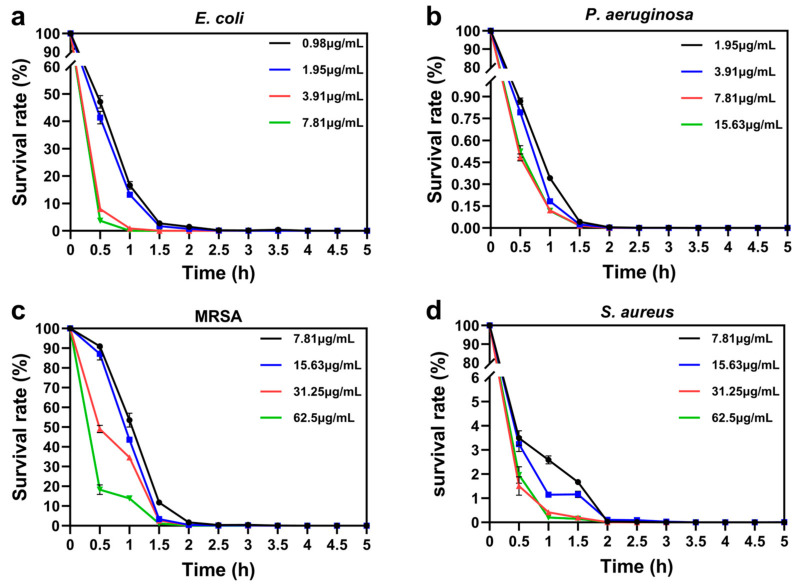
Time-dynamic curves for the survival rates of *E. coli* (**a**), *P. aeruginosa* (**b**), MRSA (**c**), and *S. aureus* (**d**) treated by different concentrations of GO@PEI-PFIL-Ag^+^/Ag/AgBr.

Bacterial survival is negatively correlated with bactericidal efficacy, and killing bacteria fast will greatly help decrease the prevalence of drug resistance. Bactericidal kinetics against the tested four strains were determined in the presence of GO@PEI-PFIL-Ag^+^/Ag/AgBr at concentrations of 1×, 2×, 4×, and 8 × MIC, as shown in Figure 6. In general, there was a decreasing trend in bacterial survival with increasing GO@PEI-PFIL-Ag^+^/Ag/AgBr concentration, contact time, or both. Bacterial survival for all the tested strains decreased to 0% within 2.5 h and remained stable, suggesting excellent bactericidal kinetics. The time to achieve a certain bactericidal rate varied with the concentration of GO@PEI-PFIL-Ag^+^/Ag/AgBr and the nature of the strains. Tables S5–S8 show the time required to reach survival rate of 50%, 10%, and 1% for the four bacteria treated with GO@PEI-PFIL-Ag^+^/Ag/AgBr at different concentrations. Again, it is worth noting that GO@PEI-PFIL-Ag^+^/Ag/AgBr at a concentration of 1 × MIC exhibited the most pronounced time-killing efficacy against Gram-negative *P. aeruginosa*, in less than 0.5 h. As for *S. aureus*, it required 1 h to reach a 1% survival rate at a higher concentration of GO@PEI-PFIL-Ag^+^/Ag/AgBr (4 × MIC). Slight toughness was encountered in killing *E. coli* and MRSA. When *E. coli* and MRSA were treated with GO@PEI-PFIL-Ag^+^/Ag/AgBr at 1 × MIC, it required spending 1 h and 1.5 h, respectively, to reach a 50% survival rate. However, these two bacteria are more sensitive to the concentration of GO@PEI-PFIL-Ag^+^/Ag/AgBr, and a higher concentration of the composite could decrease the survival rate to 50% within 0.5 h. The comparison of the results with the four bacteria revealed that the composites were more effective in killing Gram-negative bacteria than Gram-positive bacteria, which may be due to their different cell surface structures and compositions [85]. The peptidoglycan layer of Gram-positive bacteria, which is many times thicker than that of Gram-negative bacteria, may have slowed down the destructive effect of GO@PEI-PFIL-Ag^+^/Ag/AgBr, whereas GO@PEI-PFIL-Ag^+^/Ag/AgBr may more readily disrupt the outer membranes and the thin peptidoglycan layers of Gram-negative bacteria. Considering the relationship between treatment time, nanocomplex concentration, and the corresponding antimicrobial effect, we concluded that 7.81 μg/mL is the optimal therapeutic concentration of the compound and 2.5 h is the optimal treatment time.

We also examined the antimicrobial activity of the GO@PEI-PFIL-Ag^+^/Ag/AgBr composites after repeated use. As shown in Figure S6, the antimicrobial activity of the GO@PEI-PFIL-Ag^+^/Ag/AgBr composites showed little or no decrease in antimicrobial activity against *P. aeruginosa* and *S. aureus* after five reuses at a concentration of 7.81 μg/mL. It still kills about 100% of *P. aeruginosa* and *S. aureus*, which is almost as effective as it was when it was first used, and for *E. coli* and MRSA, the bacterial reduction rate drops to about 90% and 87%, respectively. The above results indicate that GO@PEI-PFIL-Ag^+^/Ag/AgBr can effectively eliminate frequent and recurrent pathogen contamination. Based on all the above results, we conclude that the material reported in this study has excellent antimicrobial activity, long-term effectiveness, fast-killing kinetics, and reusability, which proves its great potential in effectively killing pathogens and preventing microbial contamination in the long term.

#### 3.3.4. Hemolytic Activity and Cytotoxicity of GO@PEI-PFIL-Ag^+^/Ag/AgBr

**Figure 7:**
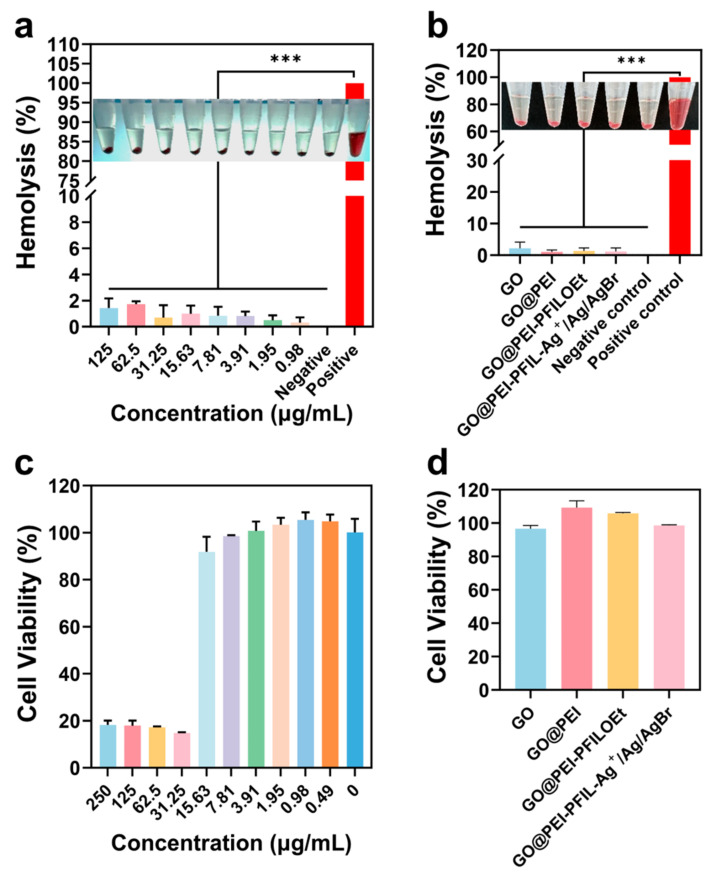
(**a**) Hemolytic activity of GO@PEI-PFIL-Ag^+^/Ag/AgBr at different concentrations against fresh human red blood cells. (**b**) Hemolytic activity of GO, GO@PEI, GO@PEI-PFILOEt and GO@PEI-PFIL-Ag^+^/Ag/AgBr at same concentrations against fresh human red blood cells. (**c**) Cytotoxicity of GO@PEI-PFIL-Ag^+^/Ag/AgBr at different concentrations against RAW264.7 cells. (**d**) Cytotoxicity of GO, GO@PEI, GO@PEI-PFILOEt, and GO@PEI-PFIL-Ag^+^/Ag/AgBr at same concentrations against RAW264.7 cells. Statistical significance was set at * *p* < 0.05, ** *p* < 0.01, and *** *p* < 0.001. All the experiments were repeated three times.

In order to evaluate the biosafety of the composite material GO@PEI-PFIL-Ag^+^/Ag/AgBr together with GO, GO@PEI, and GO@PEI-PFILOEt at 7.81 μg/mL, hemolytic activities against blood erythrocytes in healthy individuals were first performed. As shown in Figure 7b, these materials exhibited negligible hemolytic activities, proving that they have high blood biocompatibility. We then examined the impact of GO@PEI-PFIL-Ag^+^/Ag/AgBr at different concentrations on the hemolytic activities. The results, shown in Figure 7a, indicated that even at a higher concentration of 125 μg/mL (16- to 128-fold relative to the MICs for the four tested strains), the hemolysis rate of GO@PEI-PFIL-Ag^+^/Ag/AgBr was just measured at 1.4%, well below the clinically accepted value of 10%, thus it hardly caused any significant hemolysis. The row of centrifuge vials inside Figure 7a,b visualized the experimental results, proving that these materials have high blood biocompatibility. The cytotoxicity of the composites on mouse peritoneal RAW264.7 macrophages was detected by a CCK-8 assay (Figure 7c). After being treated with GO@PEI-PFIL-Ag^+^/Ag/AgBr at a concentration of 15.63 μg/mL for 24 h, RAW264.7 cells still remained at as high as 90% of viability. A further increase in concentration will decrease the cell viability below 15%. Figure 7d displayed that parental GO, GO@PEI, and GO@PEI-PFILOEt at 7.81 μg/mL were compatible to RAW264.7 cells without causing a significant decrease in cell viability.

#### 3.3.5. Inhibition and Disruption of Biofilms

**Figure 8:**
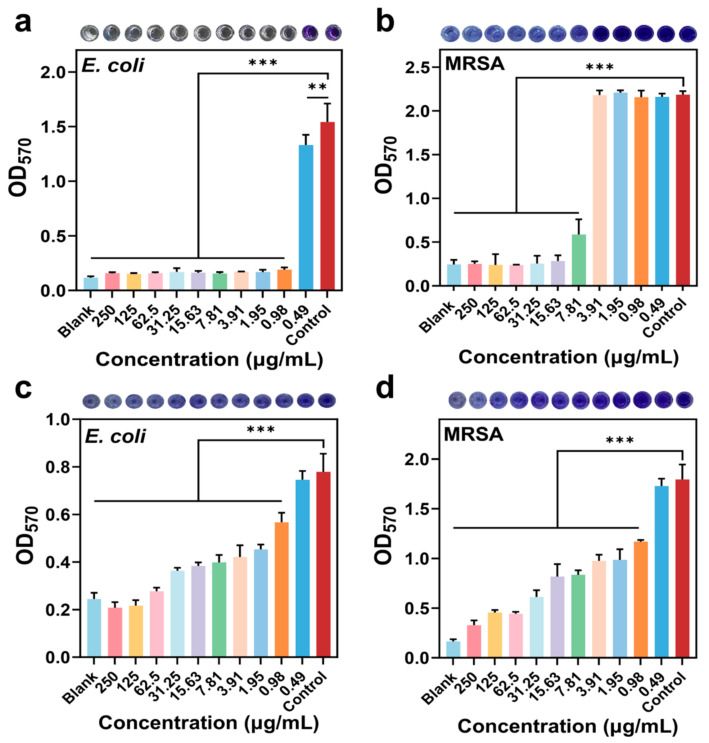
Effect of GO@PEI-PFIL-Ag^+^/Ag/AgBr on inhibition of biofilm formation in *E. coli* (**a**) and MRSA (**b**). Effect of GO@PEI-PFIL-Ag^+^/Ag/AgBr on disruption of preformed biofilms in *E. coli* (**c**) and MRSA (**d**). Statistical significance was set at * *p* < 0.05, ** *p* < 0.01, and *** *p* < 0.001. All the experiments were repeated three times.

Biofilms are extracellular polymeric substances (EPSs) consisting of a variety of nutrients (e.g., exocytotic polysaccharides, proteins, lipids, bacterial DNA, and enzymes) that surround and adhere to the surface of a material or tissue, and act as barriers to antibiotic penetration and macrophage attack, which can help bacteria to survive in hostile environments, and are a major driver of drug resistance and recurrent infections [86]. New materials are highly needed to combat bacterial biofilms. Silver nanoparticles, in combination with other active compounds, have demonstrated antibiofilm activity [87]. The effects of GO@PEI-PFIL-Ag^+^/Ag/AgBr alone on the inhibition and disruption of biofilms were investigated, with *E. coli* and MRSA as model pathogens (Figure 8). The inhibition ability was quantified by staining the bacterial biofilm with crystal violet, and the measured intensity of absorption at 570 nm was positively correlated with biofilm content. As the concentration of GO@PEI-PFIL-Ag^+^/Ag/AgBr increased, the staining of the bacterial solution became lighter, indicating reduced biofilm formation. Relative to the control group, the experimental group of *E. coli* (Figure 8a) treated by GO@PEI-PFIL-Ag^+^/Ag/AgBr at 1 × MIC showed a significant lightening of the color, which was almost equal to that of the blank control group, and the OD570 value was decreased to 88%. As for MRSA (Figure 8b), a similar trend was observed with a 73% decrease in the OD570 value. These results mean that GO@PEI-PFIL-Ag^+^/Ag/AgBr, even at a low concentration, has a potent inhibitory effect on biofilm formation of *E. coli* and MRSA. In order to elucidate which modification step contributes more to the inhibitory potency, GO, GO@PEI, and GO@PEI-PFILOEt were then tested. It was observed that compared to GO and GO@PEI, metal-free GO@PEI-PFILOEt at 7.81 μg/mL showed a greater inhibitory effect on the biofilm formation of *E. coli* (63% decrease in OD), as shown in Figure S7a. However, these three materials at 7.81 μg/mL showed no inhibitory potency against the biofilm formation of MRSA (Figure S7b).

The ability of GO@PEI-PFIL-Ag^+^/Ag/AgBr at varied concentrations to destroy the preformed biofilms was also examined. As the concentration of GO@PEI-PFIL-Ag^+^/Ag/AgBr increased, the original biofilms of *E. coli* and MRSA were disrupted more and more severely (Figure 8c,d). After treating *E. coli* and MRSA with GO@PEI-PFIL-Ag^+^/Ag/AgBr at a concentration of 7.81 μg/mL, the biofilm was disrupted by 48.8% and 53.5%, respectively. It proves that GO@PEI-PFIL-Ag^+^/Ag/AgBr has a good ability to destroy the preformed biofilms. We further investigated the biofilm-disrupting ability of the same concentrations of GO, GO@PEI, GO@PEI-PFILOEt, and GO@PEI-PFIL-Ag^+^/Ag/AgBr (Figure S7c,d). It was found that GO, GO@PEI, and GO@PEI-PFILOEt showed some ability to destroy the preformed biofilms of *E. coli* and MRSA. Overall, GO@PEI-PFIL-Ag^+^/Ag/AgBr offers the best potential promise for the treatment of patients with bacterial infections.

### 3.4. Antimicrobial Mechanisms of GO@PEI-PFIL-Ag^+^/Ag/AgBr

**Figure 9:**
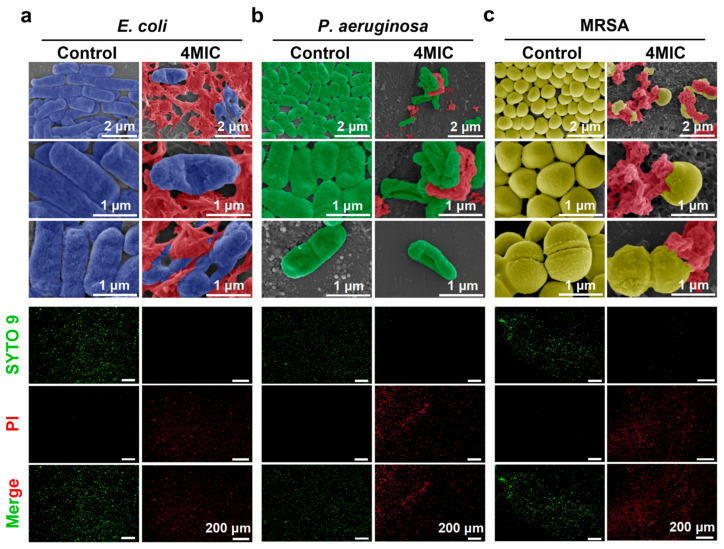
SEM analysis and live/dead fluorescent images of *E. coli* (**a**), *P. aeruginosa* (**b**), and MRSA (**c**) treated with GO@PEI-PFIL-Ag^+^/Ag/AgBr at 4 × MIC for 2.5 h.

Silver has played an important role in the history of medical advances, especially in the fight against bacterial infections [88]. Silver can be used in various forms, such as metallic bulky or nanoparticles, silver ion complexes, single component, coating, composites, or formulations. The generally accepted antibacterial mechanisms of silver materials include the disruption of bacterial cell membranes, generation of reactive oxygen species (ROS), DNA damage, and inhibition of protein synthesis [80,87,88]. In order to elucidate the antibacterial mechanism of GO@PEI-PFIL-Ag^+^/Ag/AgBr, *E. coli*, *P. aeruginosa*, and MRSA were incubated with GO@PEI-PFIL-Ag^+^/Ag/AgBr for 2.5 h, after which scanning electron microscopy was performed to observe the three types of bacteria (Figure 9). In the control group, the three bacteria were morphologically and structurally intact. *E. coli* showed a typical rod-like structure, *P. aeruginosa* also exhibited its typical structure with rounded ends and a rod-like shape, and MRSA showed a smooth spherical shape. In contrast, when exposed to GO@PEI-PFIL-Ag^+^/Ag/AgBr, the membrane structure of the three pathogens underwent very severe physical damages, such as bacterial membrane rupture openings, concave collapses, and fracture. These damages severely reduced the viability of bacteria. To further determine the disruption of bacterial cell membranes, bacterial cells were stained with SYTO 9 and PI. SYTO 9 was able to label all the bacteria, whereas the membrane-impermeant dye PI was only able to penetrate into the dead bacteria with damaged cell membranes. In the control group, *E. coli*, *P. aeruginosa*, and MRSA showed strong green fluorescence, suggesting that the bacteria retained an intact cell membrane structure. On the other hand, GO@PEI-PFIL-Ag^+^/Ag/AgBr-treated bacterial cells showed a large number of red signals, which proved that GO@PEI-PFIL-Ag^+^/Ag/AgBr could cause serious damage to the bacterial cell membranes.

**Figure 10:**
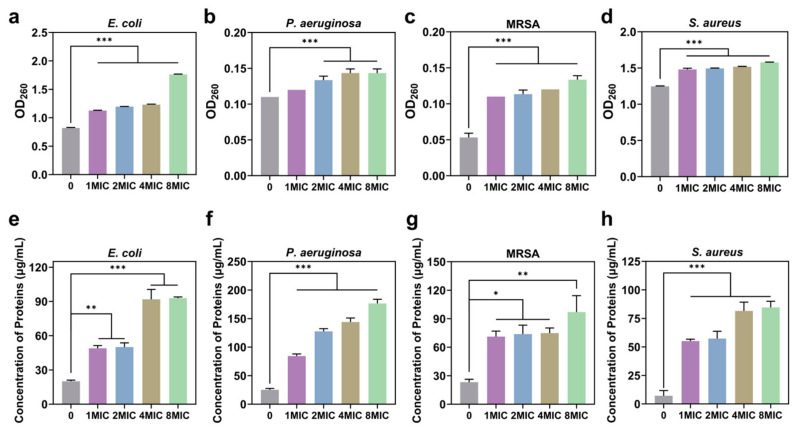
Leakage of nucleic acids from *E. coli* (**a**), *P. aeruginosa* (**b**), MRSA (**c**), and *S. aureus* (**d**) after treatment with GO@PEI-PFIL-Ag^+^/Ag/AgBr at different concentrations. Leakage of protein from *E. coli* (**e**), *P. aeruginosa* (**f**), MRSA (**g**), and *S. aureus* (**h**) after treatment with GO@PEI-PFIL-Ag^+^/Ag/AgBr at different concentrations. Statistical significance was set at * *p* < 0.05, ** *p* < 0.01, and *** *p* < 0.001. All the experiments were repeated three times.

Normally, macromolecules such as DNA and proteins do not pass in excess through the intact cell membrane of bacteria, but macromolecules can leak into the extracellular environment if the bacterial membrane is disrupted [89]. GO@PEI-PFIL-Ag^+^/Ag/AgBr can cause damage to the structure of bacterial cell membranes and alter the permeability of bacterial cell membranes, which further leads to the leakage of a variety of intracellular components, such as nucleotides and proteins, which were characterized and measured using different means. The leakage of bacterial DNA is positively correlated with the OD260 nm value. The results show (Figure 10a–d) that as the treatment concentration increases, the absorbance at 260 nm also increases, indicating that the amount of DNA leaked from the bacteria is increased. This proves that the degree of damage to the bacterial cell membrane and its permeability increase with the concentration of GO@PEI-PFIL-Ag^+^/Ag/AgBr. Additionally, it can be inferred from Figure S8 that GO, GO@PEI, GO@PEI-PFILOEt, and GO@PEI-PFIL-Ag^+^/Ag/AgBr all possess certain capabilities to disrupt cell membranes of all the tested pathogens. After the bacterial cell membrane is disrupted, not only do nucleotides leak out, but proteins also seep out. The protein content of the samples was calculated based on the standard curve plotted according to the standard protein concentration (Figure S9). As shown in Figure 10e–h, after treatment with 1×, 2×, 4×, and 8 × MIC GO@PEI-PFIL-Ag^+^/Ag/AgBr, for *E. coli*, bacterial protein leakage increased 2.4-, 2.5-, 4.5-, and 4.6-fold compared to the control. For *P. aeruginosa*, there was a 3.3-, 5.1-, 5.7-, and 7.0-fold increase over the control. For MRSA, there was a 3.1-, 3.2-, 3.2-, and 4.2-fold increase over the control group. For *S. aureus*, the most significant increases in 7.7-, 8.0-, 11.4-, and 11.9-fold were observed over the control. According to Figure S10, by successive modification on GO, the materials were increasingly capable of disrupting bacterial cell membranes. These results indicate that among all the tested materials, GO@PEI-PFIL-Ag^+^/Ag/AgBr has the strongest ability to disrupt bacterial membranes, leading to the release of intracellular substances (DNAs and proteins) into the extracellular space, thereby further accelerating bacterial death.

**Figure 11:**
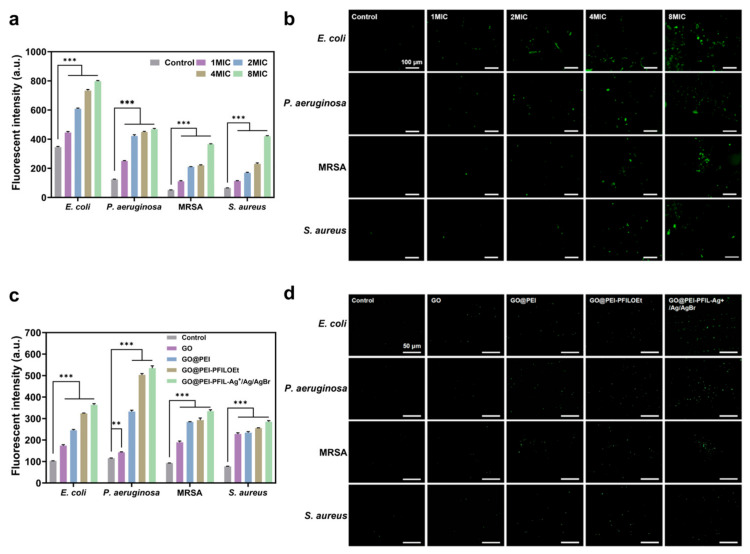
(**a**) Fluorescent intensity and (**b**) microscope images of *E. coli*, *P. aeruginosa*, MRSA, and *S. aureus* stained by DCFH-DA after being treated with GO@PEI-PFIL-Ag^+^/Ag/AgBr at different concentrations. (**c**) Fluorescent intensity and (**d**) microscope images of *E. coli*, *P. aeruginosa*, MRSA, and *S. aureus* stained by DCFH-DA after being treated with GO, GO@PEI, GO@PEI-PFILOEt, and GO@PEI-PFIL-Ag^+^/Ag/AgBr at 7.81 μg/mL. Statistical significance was set at * *p* < 0.05, ** *p* < 0.01, and *** *p* < 0.001. All the experiments were repeated three times.

In addition to disrupting the integrity of bacterial cell membranes, GO@PEI-PFIL-Ag^+^/Ag/AgBr also generated reactive oxygen species (ROS), causing cellular damage through multiple mechanisms [77]. As shown in Figure 11a, the amount of ROS produced after treatment with 1×, 2×, 4×, and 8 × MIC of GO@PEI-PFIL-Ag^+^/Ag/AgBr was concentration-dependent. For example, for *S. aureus*, the levels of ROS produced after treatment were elevated by approximately 75%, 161%, 257%, and 549%, respectively, compared to the control. The green fluorescent signal produced by ROS was further observed by fluorescence microscopy images (Figure 11b). Subsequently, Figure 11c,d show that the ability to generate ROS was progressively enhanced during the functionalization and modification of the material. For *P. aeruginosa*, from the modification of GO to GO@PEI-PFIL-Ag^+^/Ag/AgBr, the level of ROS increased by approximately 24%, 188%, 336%, and 363% compared to the control group, respectively. Maiti and Banerji recently reported that ammonium-based ionic liquid can activate molecular oxygen to generate ROS [90]. Therefore, it might be reasonable to consider that the metal-free GO@PEI-PFILOEt is responsible for the dramatic increase in ROS. Additionally, coordinated Ag(I) ions and nanoparticles of Ag/AgBr in GO@PEI-PFIL-Ag^+^/Ag/AgBr synergistically contribute to the highest level of ROS [91], and the oxidative stress damage caused by ROS leads to the apoptosis of bacterial cells [92].

Before we move to the conclusion section, it would be useful to make a concise analysis of the structure–activity relationship (SAR) in the context of the comprehensive knowledge obtained with a variety of silver-based antibacterial materials. The prepared GO@PEI-PFIL-Ag^+^/Ag/AgBr composite is endowed with antimicrobial potentiation by its structural uniqueness that combines some antibacterial elements. The high density of cations on the surface enhances the electrostatic interactions with the bacterial cell wall and membrane, which is hardly realized between the negatively charged silver nanoparticles and bacterial envelope [93]. The high loading of silver in three forms reduces the amount of materials required for effectiveness against pathogens [94], and ligand exchange helps the transportation and penetration of Ag(I) ions into the damaged cell membranes to incapacitate the internal substances.

## 4. Conclusions

In this study, we report a graphene oxide-based broad-spectrum antibacterial composite meticulously modified with phosphonate-functionalized ionic liquid (GO@PEI-PFIL-Ag^+^/Ag/AgBr). This material exhibits rapid broad-spectrum bactericidal activity against tested strains of *E. coli*, *P. aeruginosa*, MRSA, and *S. aureus*. Additionally, it demonstrates stable and superior performance in sustained bactericidal efficacy, reusability, and biofilm inhibition/disintegration, offering critical insights for future applications in treating persistent bacterial infections. The unique structure of its bactericidal components enables GO@PEI-PFIL-Ag^+^/Ag/AgBr to exert antibacterial effects through multiple mechanisms: disrupting bacterial membrane integrity, generating reactive oxygen species (ROS), and inactivating intracellular biomolecules via metal coordination—mechanisms that hold promise for combating multidrug-resistant infections. However, this study has limitations, including the lack of investigations into cytotoxicity toward human cells and in vivo antibacterial activity for translational applications, as well as the need for deeper exploration of ROS-related genes and more direct observations of bacterial structural damage. Future research will focus on optimizing this material to enhance its performance and further address the above-mentioned limitations to achieve a more comprehensive framework. Overall, the design concept and findings of this antibacterial material aim to provide new perspectives for addressing persistent and multidrug-resistant bacterial infections.

## Figures and Tables

**Table 1:** MIC values of different materials against bacteria.

	MIC Values (μg/mL)			
Material	Gram-Negative Bacteria		Gram-Positive Bacteria	
	*E. coli* ^1^	*P. aeruginosa* ^2^	MRSA ^3^	*S. aureus* ^4^
GO	>250	>250	>250	>250
GO@PEI	>250	>250	>250	>250
GO@PEI-PFILOEt	>250	>250	>250	>250
GO@PEI-PFIL-Cu^2+^	>250	>250	>250	≤250
GO@PEI-PFIL-Zn^2+^	>250	>250	>250	≤250
GO@PEI-PFIL-Fe^3+^	≤250	>250	>250	>250
GO@PEI-PFIL-Ag^+^/Ag/AgBr	≤0.98	≤1.95	≤7.81	≤7.81

^1^*E. coli* ATCC25922: *Escherichia coli*; ^2^ *P. aeruginosa* BNCC337940: *Pseudomonas aeruginosa*; ^3^ MRSA ATCC43300: Methicillin-resistant *Staphylococcus aureus*; and ^4^ *S. aureus* BNCC6538: *Staphylococcus aureus*.

## Data Availability

The original contributions presented in this study are included in the article/Supplementary Materials. Further inquiries can be directed to the corresponding authors.

## Supplementary Materials

The following supporting information can be downloaded at https://www.mdpi.com/article/10.3390/ma18081889/s1, Scheme S1: A detailed flowchart involving chemical modifications such as amidation, quaternization, and hydrolysis; Figure S1: XPS spectra of C 1s of GO@PEI-PFIL-Ag^+^/Ag/AgBr; Figure S2: Elemental mapping of GO (**a**), GO@PEI (**b**), and GO@PEI-PFILOEt (**c**); Figure S3: SEM (**a**) and EDS (**b**) analysis of GO@PEI-PFIL-Cu^2+^; Figure S4: EDS scanned on a silicon wafer of GO (**a**), GO@PEI (**b**), GO@PEI-PFILOEt (**c**), and GO@PEI-PFIL-Ag^+^/Ag/AgBr (**d**); Figure S5: Reduction percentage of *E. coli* (**a**), *P. aeruginosa* (**b**), MRSA (**c**), and *S. aureus* (**d**) treated with GO, GO@PEI, GO@PEI-PFILOEt, GO@PEI-PFIL-Cu^2+^, GO@PEI-PFIL-Zn^2+^, GO@PEI-PFIL-Fe^3+^, and GO@PEI-PFIL-Ag^+^/Ag/AgBr at same concentrations for 2.5 h; Figure S6: Antimicrobial effect of repeatedly used GO@PEI-PFIL-Ag+/Ag/AgBr expressed as the reduction percentages of bacteria; Figure S7: Effect of GO, GO@PEI, GO@PEI-PFILOEt, and GO@PEI-PFIL-Ag^+^/Ag/AgBr on inhibition of biofilm formation in *E. coli* (**a**) and MRSA (**b**). Effect of GO, GO@PEI, GO@PEI-PFILOEt, and GO@PEI-PFIL-Ag^+^/Ag/AgBr on disruption of the preformed biofilms in *E. coli* (**c**) and MRSA (**d**); Figure S8: Leakage of nucleic acids from *E. coli* (**a**), *P. aeruginosa* (**b**), MRSA (**c**), and *S. aureus* (**d**) after treatment with GO, GO@PEI, GO@PEI-PFILOEt, and GO@PEI-PFIL-Ag^+^/Ag/AgBr at 7.81 μg/mL; Figure S9: Calibration curve of protein concentration standards by Bradford; Figure S10: Leakage of proteins from *E. coli* (**a**), *P. aeruginosa* (**b**), MRSA (**c**), and *S. aureus* (**d**) after treatment with GO, GO@PEI, GO@PEI-PFILOEt, and GO@PEI-PFIL-Ag^+^/Ag/AgBr at 7.81 μg/mL; Table S1: The D and G band values and the I_D_/I_G_ ratio of GO, GO@PEI, GO@PEI-PFILOEt, and GO@PEI-PFIL-Ag^+^/Ag/AgBr; Table S2: Atomic contents of C, O, N, P, Br, and Ag in GO@PEI-PFIL-Ag+/Ag/AgBr based on the XPS measurements; Table S3: Atomic contents of C, O, N, P, Br, and Ag in GO, GO@PEI, GO@PEI-PFILOEt, and GO@PEI-PFIL-Ag+/Ag/AgBr based on the EDS measurements; Table S4: Compare several reported antibacterial materials based on graphene oxide or based on silver; Table S5: The required duration for a certain survival rate of E. coli treated with different concentrations of GO@PEI-PFIL-Ag+/Ag/AgBr; Table S6: The required duration for a certain survival rate of P. aeruginosa treated with different concentrations of GO@PEI-PFIL-Ag+/Ag/AgBr; Table S7: The required duration for a certain survival rate of MRSA treated with different concentrations of GO@PEI-PFIL-Ag+/Ag/AgBr; and Table S8: The required duration for a certain survival rate of *S. aureus* treated with different concentrations of GO@PEI-PFIL-Ag^+^/Ag/AgBr [56,66,67,68,69,70,71,72,73].
