# Supplementary material for: Antibacterial Activity of GO-Based Composites Enhanced by Phosphonate-Functionalized Ionic Liquids and Silver
Source: Materials (Basel). 2025 Apr 21;18(8):1889. doi: 10.3390/ma18081889 (PMC12028358; doi:10.3390/ma18081889)
Supplement: Supplementary file 1 [file materials-18-01889-s001.zip › materials-3584826-supplementary.pdf]

Supplementary Materials

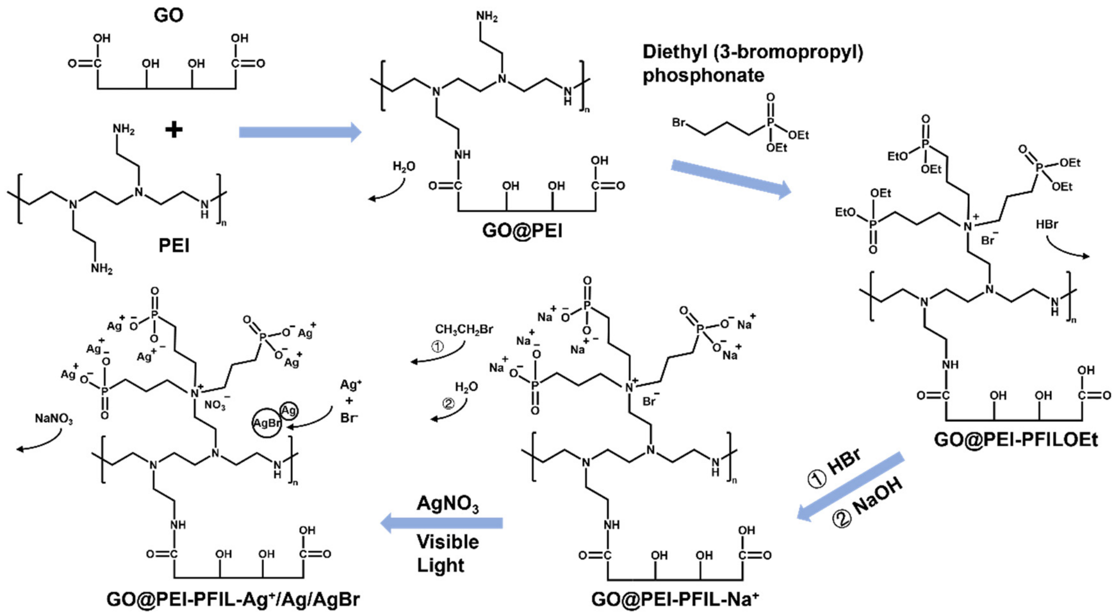

Scheme S1. A detailed flowchart involving chemical modifications such as amidation, quaternization, and hydrolysis.

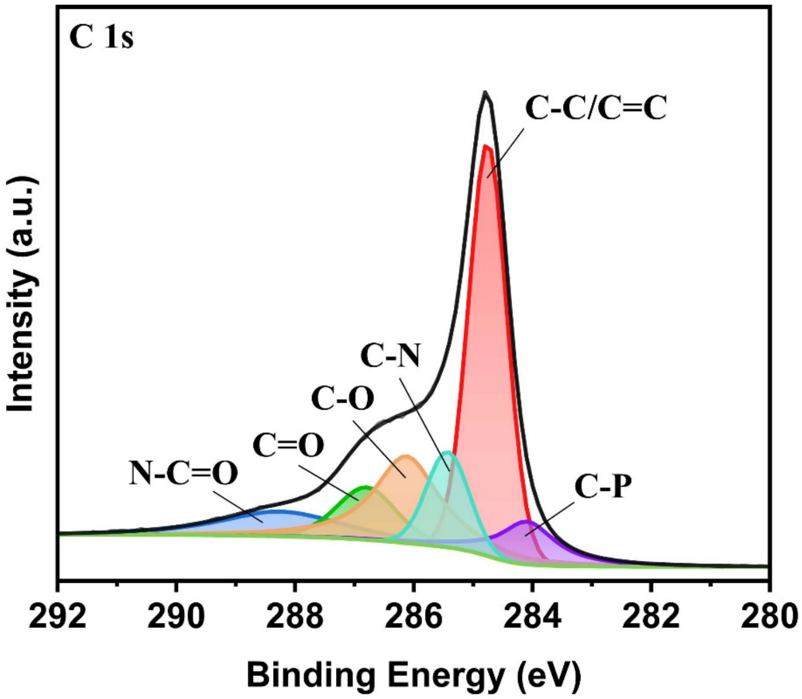

Figure S1. XPS spectra of C 1s of GO@PEI-PFIL-Ag^{+}/Ag/AgBr.

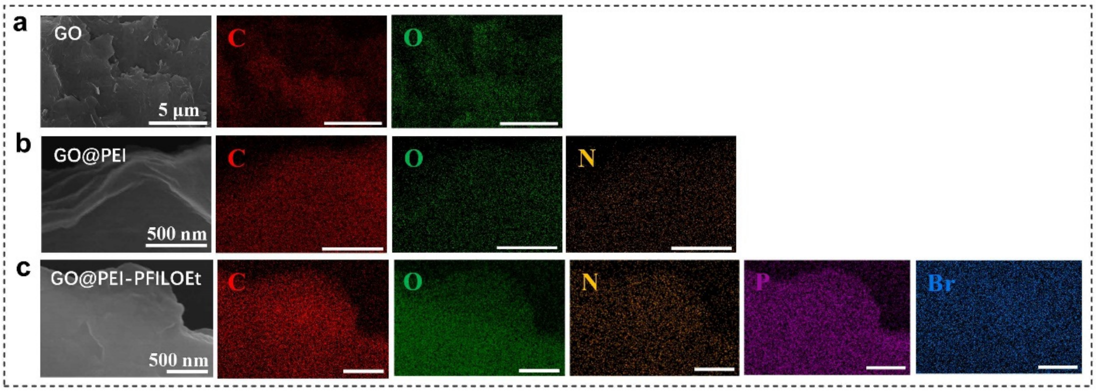

Figure S2. Elemental mapping of GO (a), GO@PEI (b) and GO@PEI-PFILOEt (c).

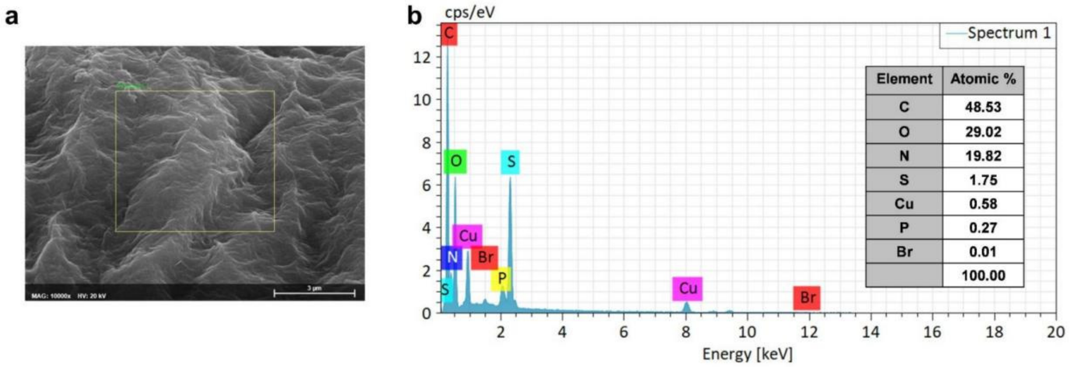

Figure S3. SEM (a) and EDS (b) analysis of GO@PEI-PFIL-Cu^{2+}.

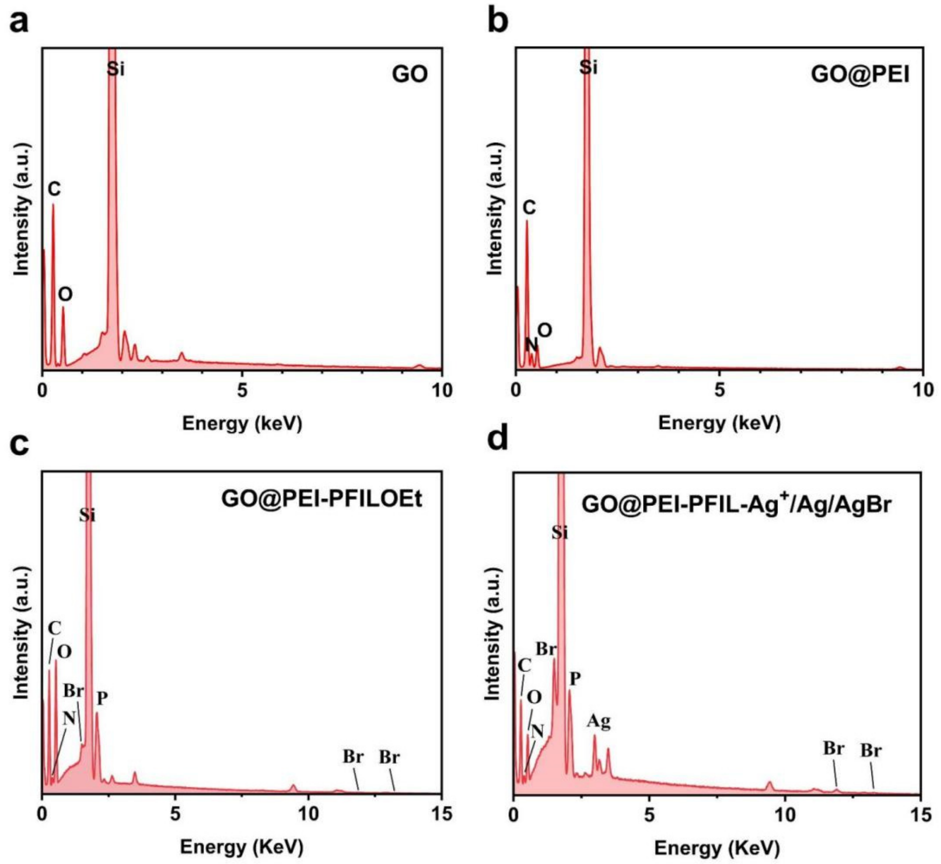

Figure S4. EDS scanned on a silicon wafer of GO (a), GO@PEI (b), GO@PEI-PFILOEt (c) and GO@PEI-PFIL-Ag^+/Ag/AgBr (d).

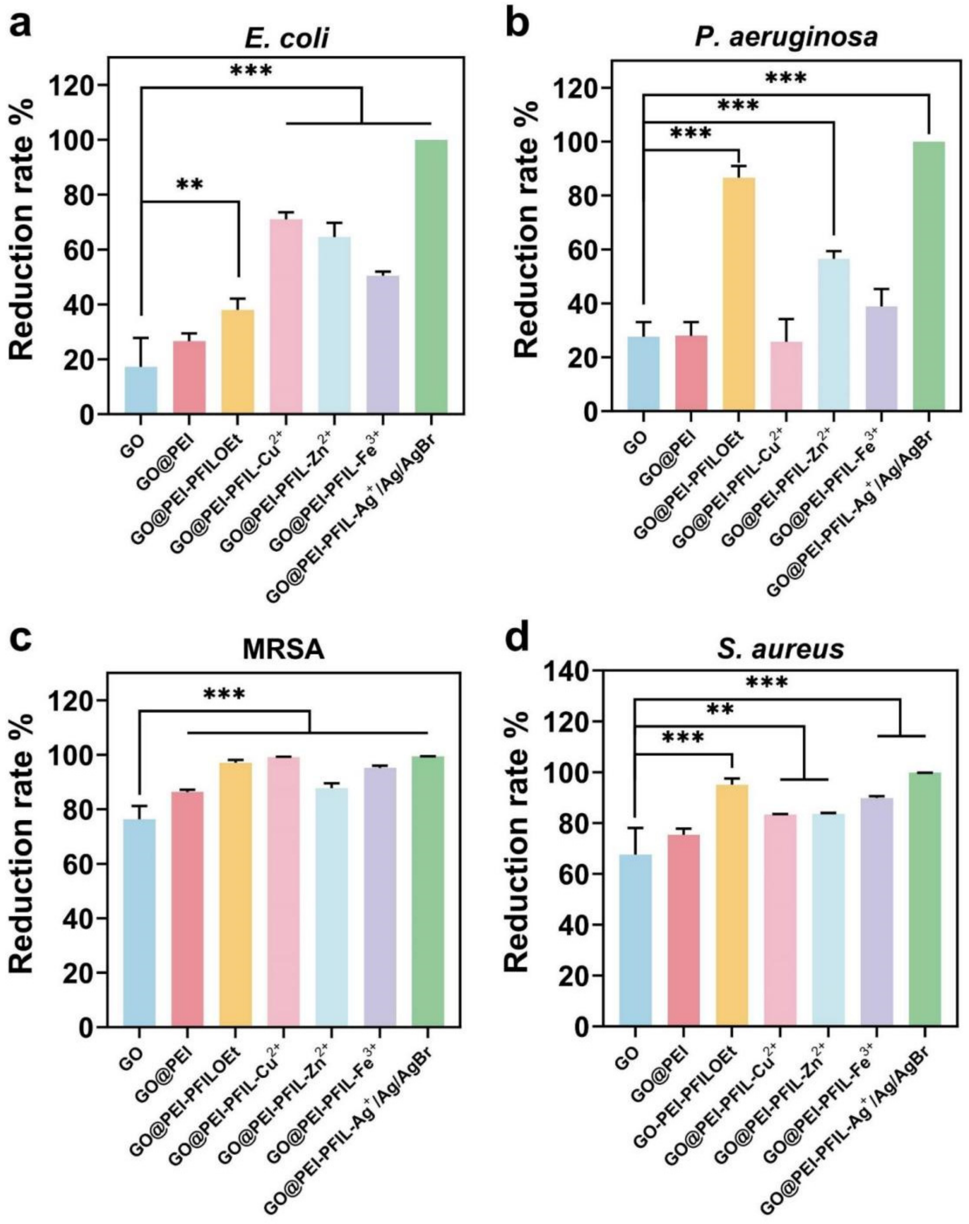

Figure S5. Reduction percentage of *E. coli* (a), *P. aeruginosa* (b), MRSA (c) and *S. aureus* (d) treated with GO, GO@PEI, GO@PEI-PFILOEt, GO@PEI-PFIL-Cu^{2+}, GO@PEI-PFIL-Zn^{2+}, GO@PEI-PFIL-Fe^{3+} and GO@PEI-PFIL-Ag^{+}/Ag/AgBr at same concentrations for 2.5 hours.

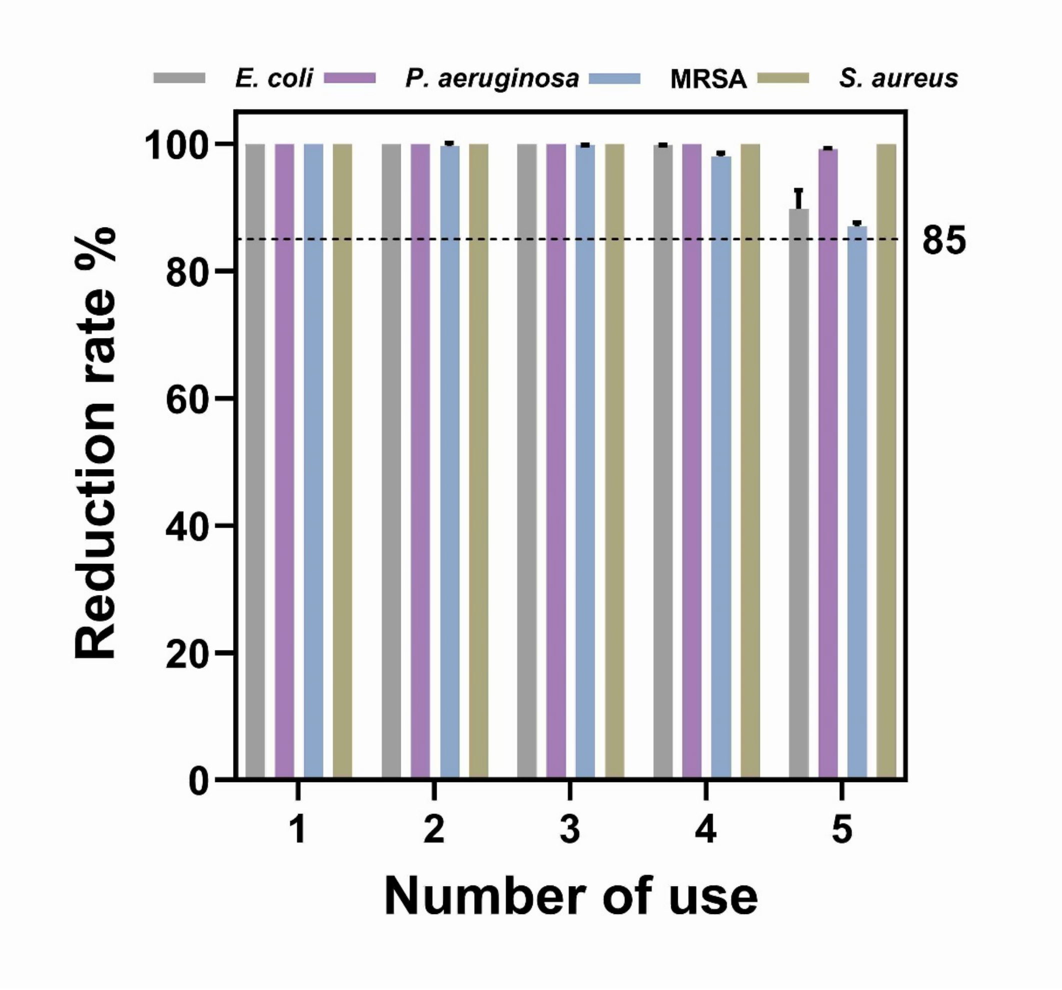

Figure S6. Antimicrobial effect of repeatedly used GO@PEI-PFIL-Ag^+/Ag/AgBr expressed as the reduction percentages of bacteria.

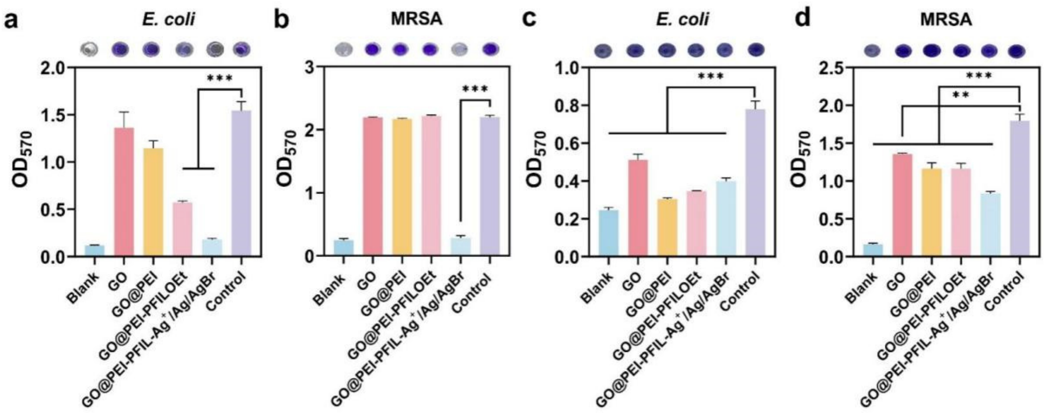

Figure S7. Effect of GO, GO@PEI, GO@PEI-PFILOEt and GO@PEI-PFIL-Ag^{+}/Ag/AgBr on inhibition of biofilm formation in *E. coli* (a) and MRSA (b). Effect of GO, GO@PEI, GO@PEI-PFILOEt and GO@PEI-PFIL-Ag^{+}/Ag/AgBr on disruption of the preformed biofilms in *E. coli* (c) and MRSA (d).

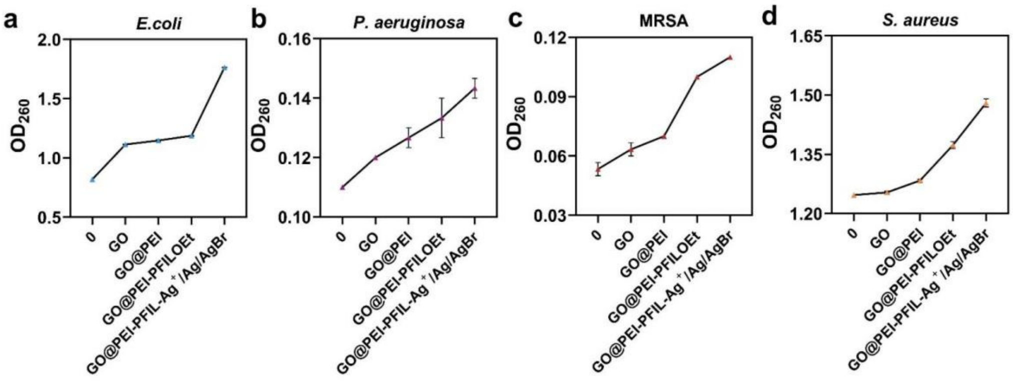

Figure S8. Leakage of nucleic acids from *E. coli* (a), *P. aeruginosa* (b), MRSA (c) and *S. aureus* (d) after treatment with GO, GO@PEI, GO@PEI-PFILOEt and GO@PEI-PFIL-Ag^+/Ag/AgBr at 7.81 μg/mL.

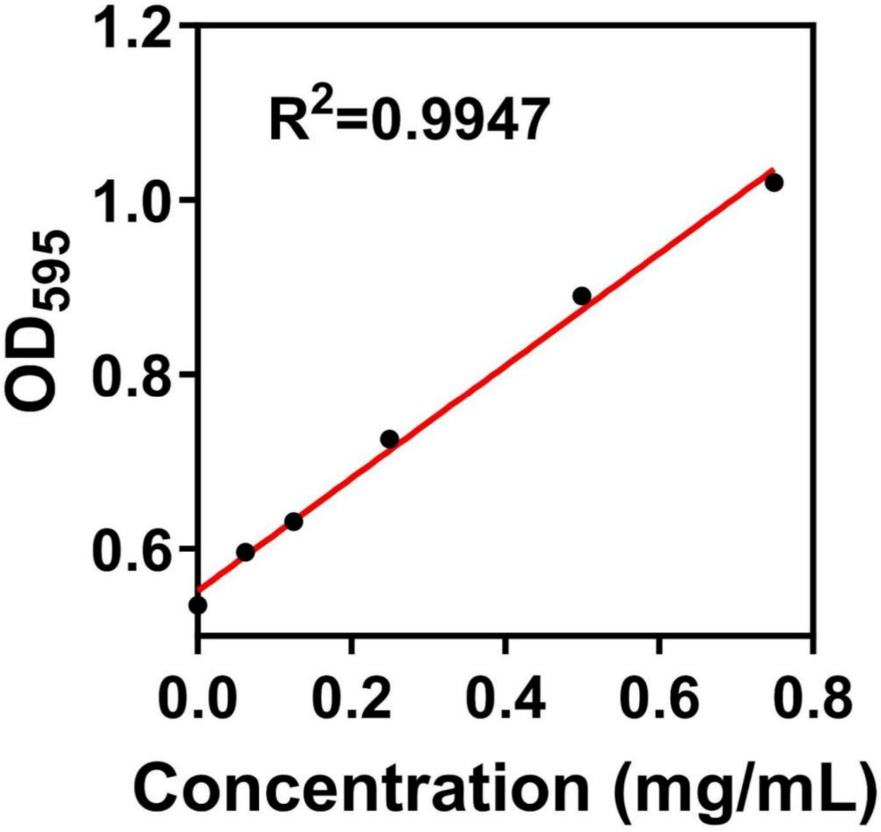

Figure S9. Calibration curve of protein concentration standards by Bradford.

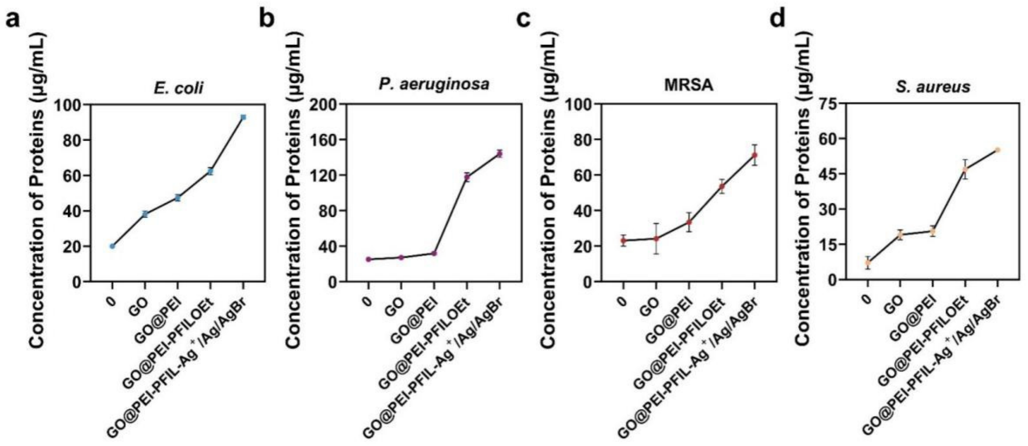

Figure S10. Leakage of proteins from *E. coli* (a), *P. aeruginosa* (b), MRSA (c) and *S. aureus* (d) after treatment with GO, GO@PEI, GO@PEI-PFILOEt and GO@PEI-PFIL-Ag^+/Ag/AgBr at 7.81 μg/mL.

Table S1. The D and G band values and the I_D/I_G ratio of GO, GO@PEI, GO@PEI-PFILOEt and GO@PEI-PFIL-Ag^+/Ag/AgBr.

Sample	GO	GO@PEI	GO@PEI-PFILOEt	GO@PEI-PFIL-Ag^+/Ag/AgBr
D band (cm^{-1})	1348	1343	1340	1348
G band (cm^{-1})	1596	1585	1581	1578
I_D/I_G	0.94	1.05	0.97	0.98

Table S2. Atomic contents of C, O, N, P, Br and Ag in GO@PEI-PFIL-Ag^{+}/Ag/AgBr based on the XPS measurements.

Element	C 1s	O 1s	N 1s	P 2p	Br 3d	Ag 3d
Atomic%	79.36	11.79	6.23	0.45	0.92	1.25

Table S3. Atomic contents of C, O, N, P, Br and Ag in GO, GO@PEI, GO@PEI-PFILOEt and GO@PEI-PFIL-Ag^+/Ag/AgBr based on the EDS measurements.

Sample/Element	C	O	N	P	Br	Ag
GO	90.44	9.56	−	−	−	−
GO@PEI	79.55	6.43	14.02	−	−	−
GO@PEI-PFILOEt	74.77	19.75	5.13	0.29	0.06	−
GO@PEI-PFIL-Ag^+/Ag/AgBr	83.73	9.17	5.49	0.2	0.52	0.89

Table S4. Comparison with several reported antibacterial materials based on graphene oxide or based on silver.

Types of antibacterial materials	Types of bacteria	$MIC_{Gram\text{-}positive\ bacteria}$ (μg/mL)	$MIC_{Gram\text{-}negative\ bacteria}$ (μg/mL)	Ref
Ag/AgBr-MBG	Broad-spectrum	10 to *S. aureus* and MRSA	10 to *E. coli*	[1]
AgNPs	Broad-spectrum	42.75 to *S. aureus*	85.51 to *E. coli*	[2]
AgBr@PBGDBr-C	Broad-spectrum	128 to *S. aureus*	64 to *E. coli*	[3]
GO-Ag_3PO_4	Gram-negative bacteria	-	20 to *E. coli*	[4]
Ag(I)-ATPS	Broad-spectrum	32.5 to *S. aureus*	25.5 to *E. coli*	[5]
GO-CS-Ag	Broad-spectrum	10 to *S. aureus*	8 to *E. coli,* 7 to *P. aeruginosa*	[6]
GO@PEG@AgNPs (30 nm)	Broad-spectrum	8 to *S. aureus*	6 to *E. coli*	[7]
AgNCs	Broad-spectrum	4 to MRSA	3 to *E. coli*	[8]
PEI-GO	Gram-positive bacteria	8 to *S. aureus* and MRSA	＞128 to *E. coli*	[9]
GO@PEI-PFIL-Ag^+/Ag/AgBr	Broad-spectrum	7.81 to *S. aureus* and MRSA	0.98 to *E. coli,* 1.95 to *P. aeruginosa*	This work

Reference for Table S4

1. Chen, W.; Wu, W.; Bai, Q.; Liu, J.; Zheng, C.; Gao, Q.; Hu, F.; Zhang, Y.; Lu, T. Photocatalytic Ag/AgBr-MBG for Rapid Antibacterial and Wound Repair. *Acs Biomater Sci Eng* **2023**, *9*, 2470-2482, doi:10.1021/acsbiomaterials.3c00039.
2. Al-Sawarees, D.K.; Darwish, R.M.; Abu-Zurayk, R.; Masri, M.A. Assessing silver nanoparticle and antimicrobial combinations for antibacterial activity and biofilm prevention on surgical sutures. *J Appl Microbiol* **2024**, *135*, doi:10.1093/jambio/lxae063.
3. Wang, B.; He, L.; Zhou, F.; Huang, J.; Yu, W.; Chen, H.; Gan, J.; Song, M.; Yang, X.; Zhu, R. Exploiting the advantages of cationic copolymers and AgBr nanoparticles to optimize the antibacterial activity of chitosan. *Int J Biol Macromol* **2024**, *270*, doi:10.1016/j.ijbiomac.2024.132209.
4. Liu, L.; Liu, J.C.; Sun, D.D. Graphene oxide enwrapped Ag3PO4 composite: towards a highly efficient and stable visible-light-induced photocatalyst for water purification. *Catal Sci Technol* **2012**, *2*, 2525-2532, doi:10.1039/c2cy20483e.
5. Alahmadi, N.S.; Elshaarawy, R.F.M. Novel aminothiazolyl-functionalized phosphonium ionic liquid as a scavenger for toxic metal ions from aqueous media; mining to useful antibiotic candidates. *J Mol Liq* **2019**, *281*, 451-460, doi:10.1016/j.molliq.2019.01.154.
6. Khawaja, H.; Zahir, E.; Asghar, M.A.; Asghar, M.A. Graphene oxide, chitosan and silver nanocomposite as a highly effective antibacterial agent against pathogenic strains. *Colloids and Surfaces A: Physicochemical and Engineering Aspects* **2018**, *555*, 246-255, doi:10.1016/j.colsurfa.2018.06.052.

7. Chen, X.; Huang, X.; Zheng, C.; Liu, Y.; Xu, T.; Liu, J. Preparation of different sized nano-silver loaded on functionalized graphene oxide with highly effective antibacterial properties. *J Mater Chem B* **2015**, *3*, 7020-7029, doi:10.1039/c5tb00280j.
8. Xie, X.; Sun, T.; Xue, J.; Miao, Z.; Yan, X.; Fang, W.; Li, Q.; Tang, R.; Lu, Y.; Tang, L.; et al. Ag Nanoparticles Cluster with pH‐Triggered Reassembly in Targeting Antimicrobial Applications. *Adv Funct Mater* **2020**, *30*, doi:10.1002/adfm.202000511.
9. Fan, Z.; Po, K.H.L.; Wong, K.K.; Chen, S.; Lau, S.P. Polyethylenimine-Modified Graphene Oxide as a Novel Antibacterial Agent and Its Synergistic Effect with Daptomycin for Methicillin-Resistant Staphylococcus aureus. *ACS Applied Nano Materials* **2018**, *1*, 1811-1818, doi:10.1021/acsanm.8b00219.

Table S5. The required duration for a certain survival rate of *E. coli* treated with different concentrations of GO@PEI-PFIL-Ag^+/Ag/AgBr.

GO@PEI-PFIL-Ag^+/Ag/AgBr concentration	The time required for *E. coli* to reach a certain viability		
	50%	10%	1%
0.98 μg/mL	0.5–1h	1–1.5h	2–2.5h
1.95 μg/mL	0.5–1h	1–1.5h	1.5–2h
3.91 μg/mL	<0.5h	<0.5h	0.5–1h
7.81 μg/mL	<0.5h	<0.5h	0.5–1h

Table S6. The required duration for a certain survival rate of *P. aeruginosa* treated with different concentrations of GO@PEI-PFIL-Ag^{+}/Ag/AgBr.

GO@PEI-PFIL-Ag^{+}/Ag/AgBr concentration	The time required for *P. aeruginosa* to reach a certain viability		
	50%	10%	1%
1.95 μg/mL	<0.5h	<0.5h	<0.5h
3.91 μg/mL	<0.5h	<0.5h	<0.5h
7.81 μg/mL	<0.5h	<0.5h	<0.5h
15.63 μg/mL	<0.5h	<0.5h	<0.5h

Table S7. The required duration for a certain survival rate of MRSA treated with different concentrations of GO@PEI-PFIL-Ag^+/Ag/AgBr.

GO@PEI-PFIL-Ag^+/Ag/AgBr concentration	The time required for MRSA to reach a certain viability		
	50%	10%	1%
7.81 μg/mL	1–1.5h	1.5–2h	2–2.5h
15.63 μg/mL	0.5–1h	1–1.5h	1.5–2h
31.25 μg/mL	<0.5h	1–1.5h	1.5–2h
62.5 μg/mL	<0.5h	1–1.5h	1.5–2h

Table S8. The required duration for a certain survival rate of *S. aureus* treated with different concentrations of GO@PEI-PFIL-Ag^{+}/Ag/AgBr.

GO@PEI-PFIL-Ag^{+}/Ag/AgBr concentration	The time required for *S. aureus* to reach a certain viability		
	50%	10%	1%
7.81 μg/mL	<0.5h	<0.5h	1.5–2h
15.63 μg/mL	<0.5h	<0.5h	1–1.5h
31.25 μg/mL	<0.5h	<0.5h	0.5–1h
62.5 μg/mL	<0.5h	<0.5h	0.5–1h
